# Information Flow through a Model of the *C. elegans* Klinotaxis Circuit

**DOI:** 10.1371/journal.pone.0140397

**Published:** 2015-10-14

**Authors:** Eduardo J. Izquierdo, Paul L. Williams, Randall D. Beer

**Affiliations:** 1 Cognitive Science Program, Indiana University, Bloomington, Indiana, United States of America; 2 School of Informatics and Computing, Indiana University, Bloomington, Indiana, United States of America; Georgia State University, UNITED STATES

## Abstract

Understanding how information about external stimuli is transformed into behavior is one of the central goals of neuroscience. Here we characterize the information flow through a complete sensorimotor circuit: from stimulus, to sensory neurons, to interneurons, to motor neurons, to muscles, to motion. Specifically, we apply a recently developed framework for quantifying information flow to a previously published ensemble of models of salt klinotaxis in the nematode worm *Caenorhabditis elegans*. Despite large variations in the neural parameters of individual circuits, we found that the overall information flow architecture circuit is remarkably consistent across the ensemble. This suggests structural connectivity is not necessarily predictive of effective connectivity. It also suggests information flow analysis captures general principles of operation for the klinotaxis circuit. In addition, information flow analysis reveals several key principles underlying how the models operate: (1) Interneuron class AIY is responsible for integrating information about positive and negative changes in concentration, and exhibits a strong left/right information asymmetry. (2) Gap junctions play a crucial role in the transfer of information responsible for the information symmetry observed in interneuron class AIZ. (3) Neck motor neuron class SMB implements an information gating mechanism that underlies the circuit’s state-dependent response. (4) The neck carries more information about small changes in concentration than about large ones, and more information about positive changes in concentration than about negative ones. Thus, not all directions of movement are equally informative for the worm. Each of these findings corresponds to hypotheses that could potentially be tested in the worm. Knowing the results of these experiments would greatly refine our understanding of the neural circuit underlying klinotaxis.

## Introduction

One of the grand challenges in neuroscience is to understand how an organism’s behavior arises from the dynamical interaction between its brain, its body and its environment. An important component of that challenge involves characterizing the flow and transformation of information through a complete neural circuit, from environmental stimuli, through sensory cells, through multiple recurrent layers of interneurons and motor neurons, and finally through muscles to produce behavior. Information theory [1, 2] has become an increasingly essential tool in neuroscience, with applications ranging from studies of neural coding [3, 4] and the statistical structure of environmental stimuli [5, 6], to developing maps of functional connectivity in nervous systems [7–11]. However, there has not yet been an attempt to analyze the information flow through an entire sensorimotor circuit underlying a particular behavior. The obstacles to such an endeavor are both theoretical and experimental.

On the theoretical side, the primary challenge is how to track the dynamic flow of information. Our approach to information flow analysis incorporates several recent extensions to the basic framework of information theory [12, 13]. First, motivated by the observation that standard information measures average across all measurement outcomes, we utilize a measure of specific information that quantifies the information that components of the neural circuit provide about each specific value of the external stimulus [14–21]. Second, we apply measures of dynamic information to track how information is gained and lost by individual components and transferred between components of the neural circuit over time [22–25]. Third and finally, we treat each component of the circuit as a random process, or time-indexed sequence of random variables, and characterize how information is carried by individual components and transferred between components over time. This extension is motivated by the observation that standard information measures, and even dynamic information measures like transfer entropy [26–28], are typically treated as atemporal; for example, transfer entropy is used to quantify the information that one random process *X* at time *t* transfers to a second random process *Y* at time *t*+1 by averaging over all time indices. Instead, we consider the information at each time step to perform a more fine-grained analysis of the temporal structure of information flow (for a related approach see [22, 29, 30]).

Experimentally, there are two primary challenges. First, complete sensorimotor circuits underlying particular behaviors are rarely known. In part, this challenge can be addressed by focusing on simpler invertebrates. The nematode worm *Caenorhabditis elegans*, which has one of the simplest, most consistent and well-studied nervous systems, is uniquely qualified in this regard. The complete wiring diagram of its nervous system is known [31, 32], it is very well-characterized genetically [33, 34], it exhibits a rich behavioral repertoire [35–37], and putative circuits for several of these behaviors have been identified [38]. Second, although recent progress has been made on methods that allow simultaneous imaging of many neurons in *C. elegans* [39], collecting the magnitude of data necessary to accurately estimate all of the time-varying information measures is still difficult. We address this challenge by performing the analysis on a previously published computational model of a putative circuit for one of the nematode’s behaviors [40], since such models can be rerun any number of times under varying conditions and all relevant variables can be simultaneously recorded.

To demonstrate the utility of this approach, we focus here on salt klinotaxis, a form of chemotaxis in *C. elegans*. Klinotaxis involves gradual changes in orientation directed towards the source [41], and provides a particularly interesting behavior to analyze for two main reasons. First, although the nematode detects sensory increases and decreases in concentration primarily in different cells [42], the circuit must combine information about the full spectrum of changes in concentration in order to steer gradually towards the source. Second, klinotaxis requires state-dependence: the circuit must respond to changes in concentration differently depending on the direction of its head swing and body posture, thus combining information from the environment with its own internal state to produce an appropriate response [43]. *C. elegans* also exhibits klinotaxis using sensory stimuli other than taste, including temperature [44], odors [45], and electric fields [46]. Other species, such as the larvae of *Drosophila melanogaster*, are also known to utilize klinotaxis for spatial orientation [47, 48]. A similar strategy has also been studied in humans following a scent trail while constrained to sample the environment through a single point using a nasal prism [49]. Thus, klinotaxis may be a representative example of state-dependent spatial navigation.

**Fig 1:**
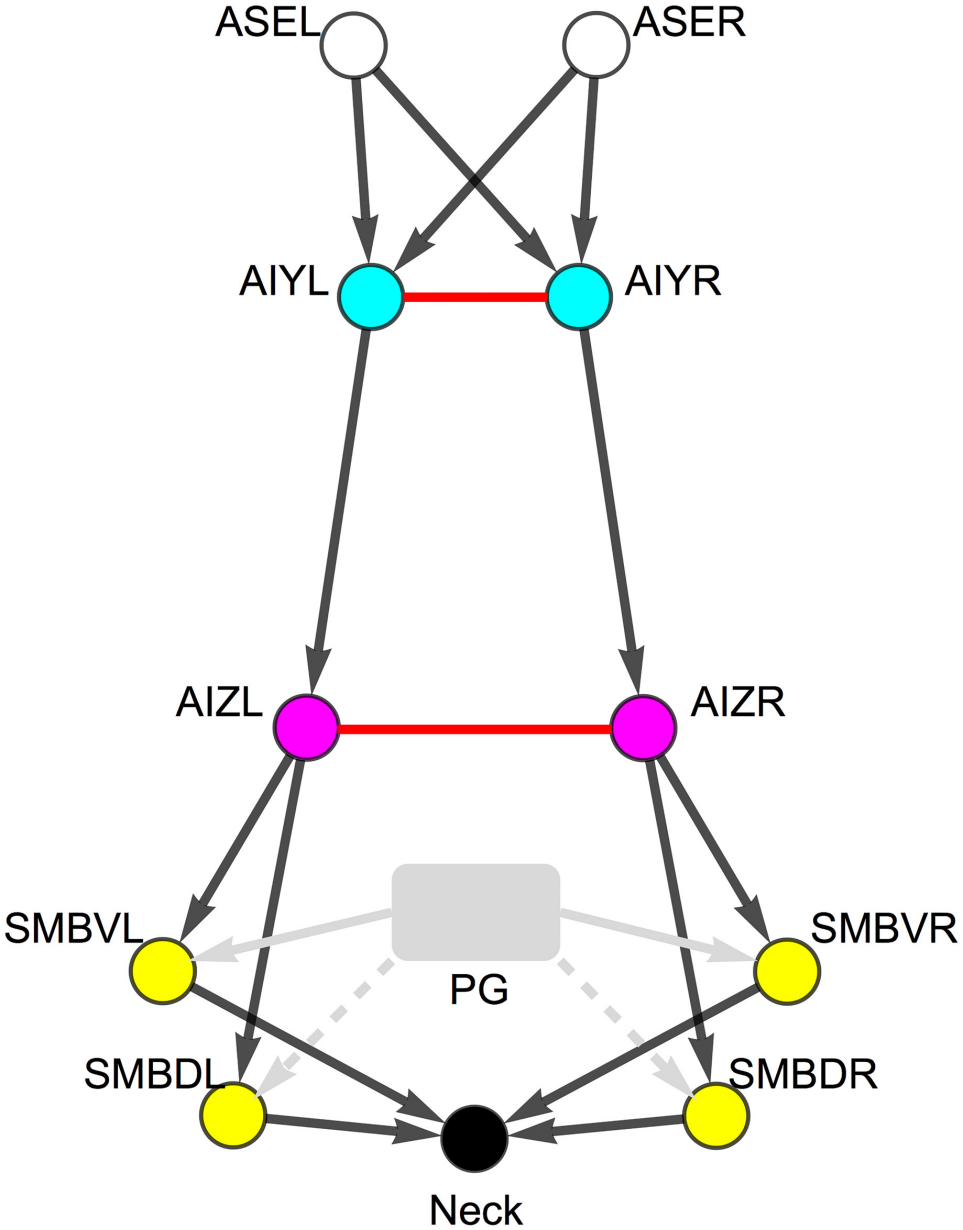
Putative minimal *C. elegans* klinotaxis circuit (adapted from [40]). Chemosensory class, ASE (white). Interneuron classes: AIY (blue) and AIZ (red). Neck motor neuron class: SMB (yellow). Neck angle (black). All classes have left and right cells. Motor neurons have additional dorsal and ventral pairs of cells. Chemical synapses shown as black arrows. Gap junctions shown as red undirected connections. Motor neurons receive an oscillatory input from a pattern generator (gray). The pattern could be generated either through proprioceptive feedback or from a central pattern generator (see Methods). The oscillatory input is antiphase for ventral (solid) and dorsal (dashed) motor neurons.

The informational analysis is performed on a previously published model of the salt klinotaxis circuit in *C. elegans* [40]. The model is grounded in the available neuroanatomical [31, 32], neurophysiological [42], and behavioral data [41]. The circuit involves four neuron classes: ASE, AIY, AIZ, and SMB; and the chemical synapses and gap junctions between them (Fig 1). The unknown electrophysiological parameters of the model, including the sign and strength of the connections, were optimized by running a large set of evolutionary searches aiming to reproduce the worm’s behavior. The result of the evolutionary searches was an ensemble of computational models of klinotaxis, which were analyzed through a combination of parameter studies and dynamical systems analysis in a previous paper [40].

An information flow analysis of a simple but complete model sensorimotor circuit allows us to engage two important theoretical issues in neuroscience. The first issue is understanding the relationship between the information flow and the underlying electrophysiology of the circuit. Generating time series recordings from the activity of cells in intact organisms is becoming increasingly common under a number of different conditions. Remarkably, in *C. elegans* this includes whole-animal imaging [39] and imaging during freely-moving behavior [50, 51]. Such time series data is exactly what is required for information theoretic analysis. And thus, such analysis is likely to become increasingly common. However, despite progress in mapping the *C. elegans* connectome [31], a characterization of the relevant biophysical properties, including voltage-gated channels, synaptic properties, and neuromodulators, lags behind. Because we have an ensemble of models of the klinotaxis circuit with known parameters, and from which we can easily generate time-series recordings under any condition, we can explore the relationship between information flow and our mechanistic understanding of the models derived from knowledge of their neurophysiological parameters.

Even if complete biophysical knowledge about the nervous system were available, the problem of understanding the general principles by which it operates would remain unaddressed. This is the second theoretical issue that an information flow analysis of a complete sensorimotor model allows us to engage. In addition to the accelerated acquisition of time series data from cellular recordings, there is also considerable effort being invested in characterizing the biophysical properties of the nervous systems of a range of model organisms. What remains unclear is how to combine these large and diverse data to arrive at principles for how an organism generates any one specific behavior from the dynamical interaction between its brain, its body and its environment. This is particularly challenging in light of the individual variability observed in circuit properties [52–54]. Because the focus of information flow analysis is on the relationship between the activity of the circuit over time and certain behaviorally-relevant features of the environment, information flow analysis has the potential to uncover higher-level descriptions of the circuit’s operation. Furthermore, because we have access to an ensemble of model circuits, we can explore the possibility that information flow analysis captures general properties of the operation of many different klinotaxis circuits, despite substantial variations in the physiological properties of those circuits.

In addition to these two broader issues, information flow analysis also allows us to address a number of specific questions about the neural basis of *C. elegans* klinotaxis. For example, where does the circuit integrate information about positive and negative changes in concentration to produce a unified action? What role do the gap junctions play in the operation of the circuit? How does the circuit combine information from the environment with its own internal state to steer in the correct direction? As information flows through the circuit, information about certain changes in concentration are preserved better than others. What specific information does the worm use to steer? We proceed in three phases. We first examine the overall flow of information about changes in salt concentration through the best model klinotaxis circuit in our ensemble. We then analyze in detail each layer of the circuit, considering the specific information carried by each cell and the pathways along which information is transferred between cells. Finally, we look broadly at the similarities and differences between the best klinotaxis circuit and the rest of the model ensemble.

## Methods

### Model

In order to characterize information flow in a simple but complete sensorimotor circuit, the information analysis in this study was performed on a previously published model of salt klinotaxis in *C. elegans* [40]. The model consists of the putative minimal klinotaxis circuit (Fig 1) connecting the main salt chemosensory neuron class ASE [42] to the neck motor class involved in modulating the amplitude of the sinusoidal locomotion, SMB [55]. The circuit was identified by mining the *C. elegans* connectome [31, 56] and constraining it using existing experimental and theoretical considerations [40]. Chemosensory neurons were modeled as idealized ON (Eq 1) and OFF (Eq 2) cells using an instantaneous function of a derivative operator applied to the recent history of attractant concentration (Eq 3) [57]. Interneurons were modeled as passive, isopotential nodes (Eqs 4 and 5). The model includes chemical synapses and electrical gap junctions. Chemical synapses were modeled as a sigmoidal function of presynaptic voltage, *σ*(*x*) = 1/(1+*e* ^−*x*^) [58]. Gap junctions were modeled as a nonrectifying conductance between two cells [59]. Neck motor neurons were modeled as passive, isopotential nodes with self-connections representing the voltage dependence of inward currents (Eq 6) [60], and an additional input from an oscillatory component (Eq 7). The model worm moves forward in undulatory fashion (Eq 8), driven by the dorsal and ventral motor neurons. The worm is represented as a single point (*x*, *y*) with instantaneous velocity, *v* (Eq 9). Altogether, the model is specified by the following set of equations:

$$\begin{array}{c} V_{\text{ON}}\left(t\right)=\{\begin{array}{cc} d(t), & \text{if}\;d(t)>0.\\ 0, & \text{otherwise}. \end{array} \end{array}$$ (1)

$$\begin{array}{c} V_{\text{OFF}}\left(t\right)=\{\begin{array}{cc} 0, & \text{if}\;d(t)>0.\\ -d(t), & \text{otherwise}. \end{array} \end{array}$$ (2)

$$\begin{array}{c} d\left(t\right)=\frac{\sum_{t-N}^{t}c\left(t\right)}{N}-\frac{\sum_{t-(N+M)}^{t-N}c\left(t\right)}{M} \end{array}$$ (3)

$$\begin{array}{c} \tau \frac{dV_{\text{AIYL}}}{dt}=-V_{\text{AIYL}}+w_{\text{(ON, AIYL)}}V_{\text{ON}}+w_{\text{(OFF, AIYL)}}V_{\text{OFF}}+g_{\text{AIY}}\left(V_{\text{AIYR}}-V_{\text{AIYL}}\right) \end{array}$$ (4)

$$\begin{array}{c} \tau \frac{dV_{\text{AIZL}}}{dt}=-V_{\text{AIZL}}+w_{\text{(AIYL, AIZL)}}\sigma \left(V_{\text{AIYL}}+\theta_{\text{AIYL}}\right)+g_{\text{AIZ}}\left(V_{\text{AIZR}}-V_{\text{AIZL}}\right) \end{array}$$ (5)

$$\begin{array}{c} \begin{array}{cc} \tau \frac{dV_{\text{SMBDL}}}{dt}=-V_{\text{SMBDL}} & +w_{\text{(AIZL,}\;\text{SMBDL)}}\sigma \left(V_{\text{AIZL}}+\theta_{\text{AIZL}}\right)\\ & +w_{\text{(SMBDL, SMBDL)}}\sigma \left(V_{\text{SMBDL}}+\theta_{\text{SMBDL}}\right)+w_{\text{PG}}V_{\text{PG}} \end{array} \end{array}$$ (6)

$$\begin{array}{c} V_{\text{PG}}=\text{sin}\left(2\pi t/T\right) \end{array}$$ (7)

$$\begin{array}{c} \begin{array}{c} \phi =\frac{d\mu }{dt}=w_{\text{NMJ}}(\left(\sigma \left(V_{\text{SMBDL}}+\theta_{\text{SMBDL}}\right)+\sigma \left(V_{\text{SMBDR}}+\theta_{\text{SMBDR}}\right)\right)-\\ \left(\sigma \left(V_{\text{SMBVL}}+\theta_{\text{SMBVL}}\right)+\sigma \left(V_{\text{SMBVR}}+\theta_{\text{SMBVR}}\right))\right) \end{array} \end{array}$$ (8)

$$\begin{array}{c} \vec{v}\left(t\right)=(\frac{dx}{dt},\frac{dy}{dt})=(v\text{cos}(\mu (t)),v\text{sin}(\mu (t))) \end{array}$$ (9)

where *c*(*t*) is the concentration at time *t*; *N* and *M* are the durations of the two intervals over which the concentration is averaged, referred to as the “rise time” and “decay time” of the sensory neurons, respectively; *V*_*i*_ represents the membrane potential for neuron *i*; *τ* is a time constant; *σ*(*x*) is the synaptic transfer function or output of the neuron; *θ*_*i*_ is a bias term for neuron *i*, which shifts the range of sensitivity of the output function; *w*_(*j*, *i*)_ represents the strength of the chemical synapse from neuron *j* to neuron *i*; *g*_*k*_ represents the conductance between neurons in class *k* (*g*_*k*_ > 0); *w*_PG_ represents the strength of the connection from the pattern generator; *T* represents the duration of a one cycle of locomotion on agar (*T* = 4.2sec) [61]; *w*_NMJ_ is the strength of the connection from motor neurons to muscles; and *v* is a constant speed of 0.022 cm/s [61]. Interneuron classes AIY and AIZ have a left and a right neuron each. Motoneuron class SMB has four neurons. Only the equation for one of the neurons in each layer is shown. The unknown parameters of the circuit were evolved using a genetic algorithm to perform klinotaxis behavior when embodied and situated. The parameters of the model were constrained to dorsal/ventral, but not left/right, symmetry. The complete procedure for developing the model, evolving the unknown parameters, and selecting the successful individuals in the population are described in [40]. The analysis in this paper focuses on that ensemble of successful circuits, including the best circuit.

### Information Theoretic Analysis

In our previous work [40], we analyzed the evolved solutions using dynamical systems theory. In this paper, we are interested in measuring the amount of information each of the components in the model has about the behaviorally relevant variable: change in concentration.

Information theoretic measurements are often applied to systems with noise, where the probability distribution is derived from repeated recordings. In a deterministic system, a probability distribution over the variables of the system can be induced by applying a distribution over the input of the system. For our analysis, we generated probability distributions by evaluating the circuit’s behavior for a sample of changes in concentration, recording the trajectories of all neural and bodily state variables for each stimulus presentation. From the values taken on by each state variable at each moment in time and the corresponding stimuli that produced them, we estimated a time-varying joint probability distribution *p*_*t*_(*s*, *r*) over values of the stimulus feature (*s*) and the response of each state variable (*r*).

The informational analysis of the circuit was performed in an open-loop configuration: we isolated the model organism from the environment, applied stimuli and measured the circuit’s response over time. We studied the system using two different assays. The first assay, called the *concentration step assay*, involved giving the circuit a step in concentration, Δ*c*, at a specific time, a method that is common experimentally [42, 60, 62, 63]. The second assay, called the *information clamp assay*, involved giving the circuit a constant change in concentration, *ċ*, for the entire stimulus duration. Unlike sensory neurons and interneurons, SMB motor neurons receive a sinusoidal input that represents the pattern generator driving the undulatory wave of locomotion. Therefore, the information response profiles for the motor neurons are time-dependent. As ASE responds to changes in concentration, a constant change in concentration means that ASE produces a constant output. The information clamp assay, thus, allows us to explore the specific role played by the sinusoidal input in regulating the flow of information in the SMB neck motor neurons.

For the main informational analysis, the sample used to generate the probability distribution for each variable was chosen from a uniform distribution, calibrated to the ranges experienced by the model worm during simulated klinotaxis (between ±0.01). We chose a uniform distribution because it is the distribution that has been applied experimentally to the study of the chemosensory neurons in the klinotaxis circuit [42]. We also analyzed the flow of information in the circuit using the empirical distribution of stimulus that the model receives during klinotaxis behavior. The empirical distribution was generated from repeated simulations.

As the model is continuous, in order to use discrete information theory we discretized the measurements. The time-varying probability distributions were estimated over a fine grid of possible stimulus values and synaptic output values. For the analysis, we used 2200 stimulus presentations, and a grid of 50 bins for the synaptic output values. We also used a kernel density estimation technique known as average shifted histograms [64], with 12 shifts along each dimension. The resulting quantities of information depend on bin size, which is typically determined using the noise floor from the recordings of the system. For the purposes of our analysis, we were only interested in the qualitative properties of the information analysis. Therefore, we verified that the results of our analysis were qualitatively robust (i.e., the overall pattern of the flow was preserved, including the relative orderings of magnitudes and the locations of the minima and maxima) over a wide range of grid resolutions (from 20 to 200 bins).

Typically information is measured in bits. Since we are concerned here with comparing how much information different components in the system have about the same random variable (i.e., the stimulus), all informational quantities were normalized by the entropy of the change in concentration sampled, *H*(*S*) = −∑_*i*_ *p*(*s*_*i*_) log *p*(*s*_*i*_). Therefore, we obtained measures that run from 0 to 1. A measure of 0 indicates that the stimulus is completely indistinguishable from other stimuli based on knowledge of the observed component, while a measure of 1 indicates that the stimulus can be uniquely determined from knowledge of the observed component.

In order to evaluate how much information various components of the sensorimotor system have about changes in concentration, we first calculated their *mutual information*. Mutual information is a measure of the dependence between two random variables, *S* and *R*; it quantifies the amount by which a measurement on one of the variables reduces our uncertainty about the other, defined as

$$\begin{array}{c} I\left(S;R\right)=\sum_{i}\sum_{j}p\left(s_{i},r_{j}\right)\log\frac{p(s_{i},r_{j})}{p\left(s_{i}\right)p\left(r_{j}\right)} \end{array}$$ (10)

where *p*(*s*, *r*) gives the joint probability distribution of *S* and *R*; *S* corresponds to the stimulus feature, in this case the change in concentration (*ċ*, Δ*c*); and *R* corresponds to the response of the various components in the system: the membrane potential of the chemosensory cells (*V*_ON_, *V*_OFF_), the synaptic output of each of the neural cells (*σ*(*V*_*e*_ + *θ*_*e*_) for *e* ∈ {AIYL, AIYR, AIZL, AIZR, SMBDL, SMBDR, SMBVL, SMBVR}) and the worm’s neck angle (*ϕ*).

Shannon’s mutual information averages across all measurement outcomes. But we are also interested in characterizing how much information the response of a component in the system has about the different specific stimuli. In order to perform a more fine-grained analysis of informational relationships, we use a measure of *specific information* [14, 15]. Unfortunately, there is no general consensus in the literature about the best way to do this. Indeed, an infinite number of measures exist that satisfy the basic requirement of measure of specific information while differing in their details. For our analysis, we adopted the most commonly used measure of specific information in the literature [16–18],

$$\begin{array}{c} I\left(S=s_{i};R\right)=\sum_{j}p\left(r_{j}|s_{i}\right)[\log\frac{1}{p(s_{i})}-\log\frac{1}{p(s_{i}|r_{j})}] \end{array}$$ (11)

where *p*(*s*∣*r*) gives the conditional probability of *s* given *r*. This measure of specific information quantifies the expected reduction in surprise about *s* given knowledge of *R*.

We are also interested in characterizing how information travels through the circuit. In order to do this, we needed to measure the amount of information transferred from one component of the system to another. *Transfer entropy* provides a general measure of the influence that one process has on another [26], defined by:

$$\begin{array}{c} T_{Y\to X}=I\left(X_{t};Y_{t-1}|X_{t-1}\right) \end{array}$$ (12)

where *Y*_*t*_ represents the state of *Y* at time *t*, and likewise for *X*. Transfer entropy quantifies the information that the previous state of *Y* provides about the next state of *X* when conditioned on *X*’s own history. Since our model is deterministic, we consider information transfer only from the previous state of *Y* while conditioning on only the previous state of *X*.

Finally, we are interested in characterizing how information is carried by individual components and transferred between components *over time*. Standard information measures, and even dynamic information measures like transfer entropy [26–28], are typically treated as atemporal. As we generated time-varying joint probability distributions, we can easily consider these measurements over time to perform a more fine-grained analysis of the temporal structure of information flow [12].

## Results

### Overall Flow of Mutual Information

**Fig 2:**
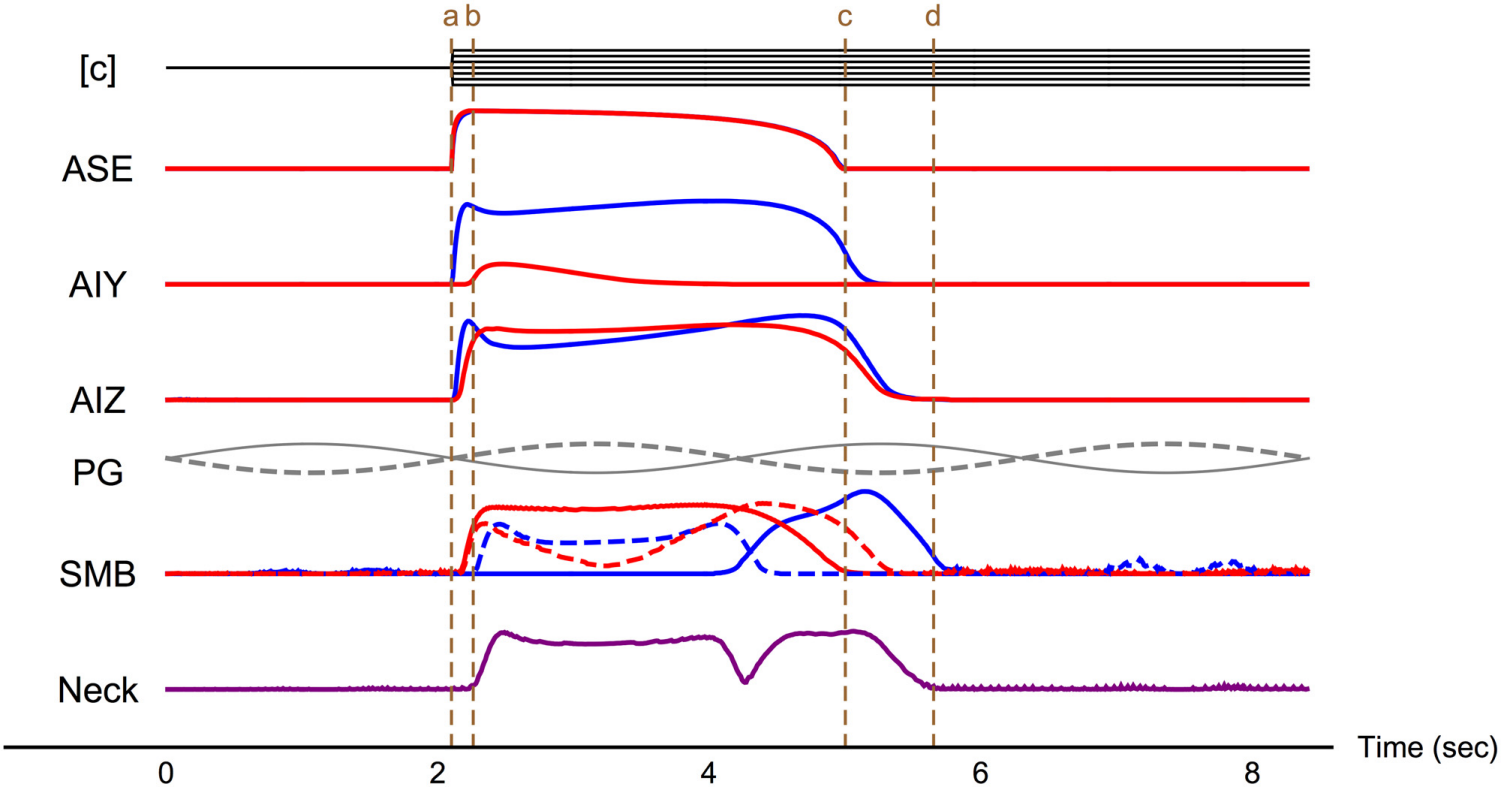
Overall flow of mutual information for concentration step assay. The top row illustrates the input to the circuit, salt concentration [c] over time. Normalized mutual information is shown for each element of the system as a function of time. For each neuron class (ASE, AIY, AIZ, SMB), the blue and red traces represent the mutual information in the left and right cells, respectively. The purple trace represents the information in the neck. The gray traces represent the oscillatory input from the pattern generator (PG) to the ventral (solid) and dorsal (dashed) motor neurons. The dashed brown lines illustrate: onset of stimulus (a), onset of neck response (b), end of sensor response to stimulus (c), and end of neck response (d). Mutual information is shown for two full locomotion cycles (8.2 secs). Example neuron output traces can be seen in Fig 6 of our previous work [40].

As a foundation for the more detailed analysis to follow, we begin with a brief overview of the overall flow of information about changes in concentration. We focus on the best performing klinotaxis circuit generated in previous work [40]. We start with the overall flow of information about the magnitude of a step change in concentration, Δ*c* (Fig 2). Specifically, we calculate the time-varying mutual information *I*(Δ*c*;*e*(*t*)) for each element *e* of the system assuming a uniform distribution over Δ*c*. We proceed by layers (Fig 1), from the chemosensory neurons (ASE), through the two layers of interneurons (AIY and AIZ), to the motor neurons (SMB), and ultimately to the neck.

The ASE class of chemosensory neurons detect Δ*c* directly. Although ASEL is only sensitive to positive values of Δ*c* and ASER is only sensitive to negative values of Δ*c*, their responses are otherwise identical: The rise in mutual information is sharp, and the information remains relatively stable for over half the locomotion cycle, after which there is a slow decay (ASE, red trace overlaps the blue trace Fig 2). Thus, despite their functional specialization, the ASE responses, viewed at the coarse level of overall mutual information, are indistinguishable.

The information response profiles for the interneurons are more interesting. Unlike for the ASE sensory neurons, the AIY interneurons exhibit a left/right asymmetry in the information that they carry about Δ*c* (AIY, Fig 2). This is somewhat surprising since both AIY neurons receive chemical synapses from both ASE neurons. Although the asymmetry in information is possible because the parameters of the model were not constrained to be left/right symmetric, asymmetry in the parameters does not limit the informational profile to be asymmetric. Even more surprising, given the information asymmetry in AIY, information is symmetric in the next layer, with AIZL and AIZR exhibiting similar information profiles (AIZ, Fig 2). Both of these informational features—asymmetry in AIY and symmetry in AIZ—turn out to be quite common across the ensemble of high-performing klinotaxis circuits. An important goal for our detailed analysis in the next section will be to examine the distinct informational pathways underlying these features.

Two additional factors complicate the informational analysis of the motor neurons (SMB, Fig 2). First, unlike the sensory neurons and interneurons, the SMB class receives a sinusoidal input representing the pattern generator that drives the undulatory wave of locomotion (PG, Fig 2). Second, there are four SMB cells arranged symmetrically about the worm’s body: dorsal-left (SMBDL), dorsal-right (SMBDR), ventral-left (SMBVL), and ventral-right (SMBVR) cells. Since the worm locomotes on its side, dorsal and ventral pairs receive anti-phase oscillatory input. For these reasons, the information response profiles for the motor neurons are complex and phase-dependent, and will be examined in detail in the next section.

Ultimately, information from all four SMB motor neurons is integrated by the body to produce a change in neck angle. Since these motor neurons are driven by the oscillatory pattern generator, we might expect the information that the neck angle carries about Δ*c* to exhibit oscillations as well. However, the information in the neck actually holds relatively constant at a high value throughout the response (Neck, Fig 2), with one exception: the information drops to essentially zero briefly around the midpoint of the locomotion cycle. This dip in information is the result of assumptions built into our model, which are discussed within the context of the detailed analysis of the neck.

How long does it take for information to travel through the system? The overview in Fig 2 highlights the various timescales of information flow within the circuit. As an approximation of the propagation time through the entire circuit, the delay from the change in concentration to the initial response of the neck is 0.16 secs (*a* to *b*, vertical dashed lines, Fig 2). Information about Δ*c* is available in the sensors for 1.9 secs (*a* to *c*), but persists in the model worm for an additional 0.65 secs after the sensory response ends (*c* to *d*), giving the approximate duration of state-dependence in the circuit. The total duration of the neck response is 3.4 secs (*b* to *d*), which corresponds to about 3/4 of a locomotion cycle. These informational timescales play an important role in understanding how this embodied circuit achieves reliable orientation when situated in a chemical environment.

**Fig 3:**
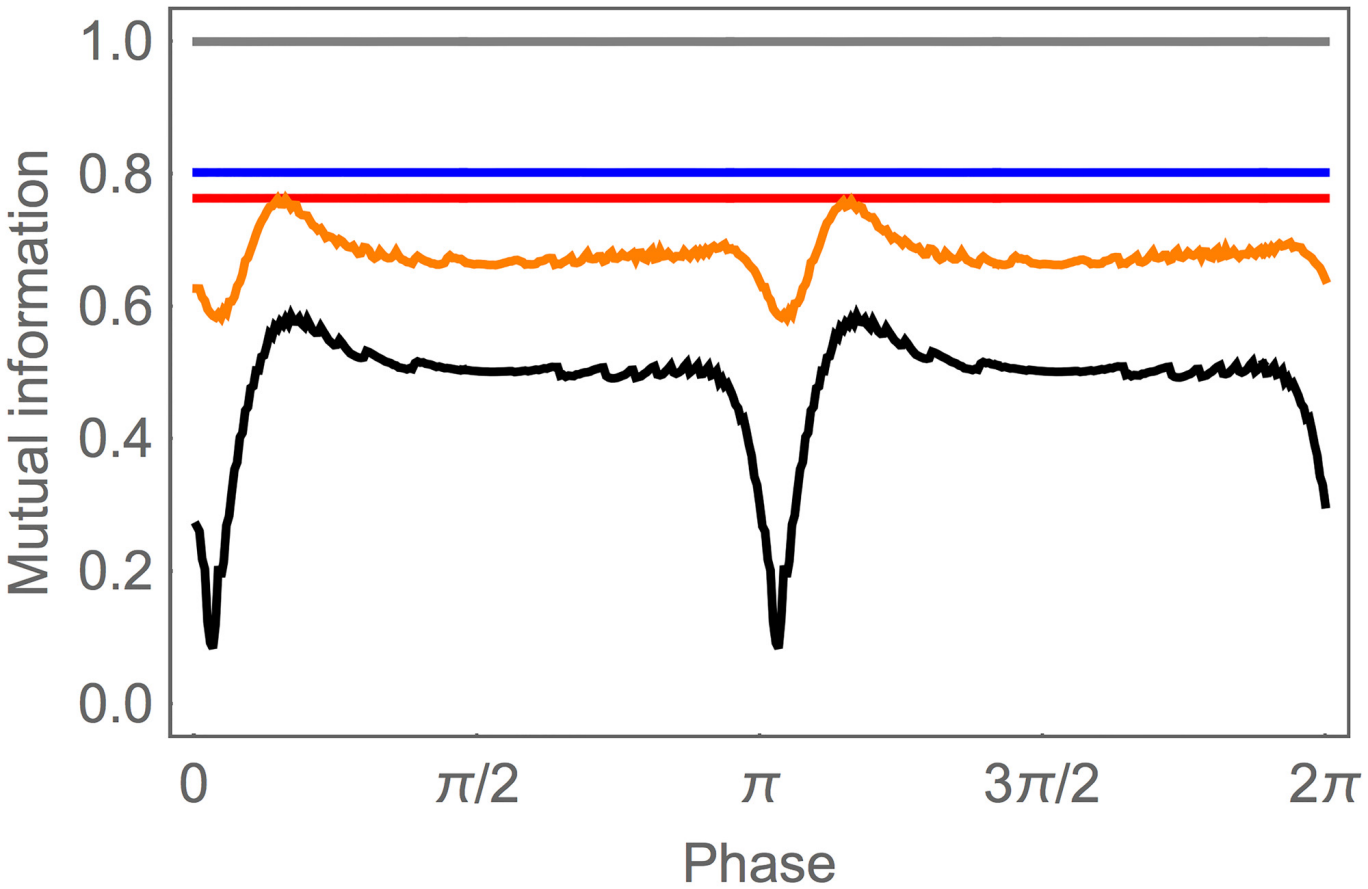
Information preservation during information clamp assay. Mutual information is shown for each of the classes in the network: ASE (gray), AIY (blue), AIZ (red), SMB (orange), and neck angle (black). Traces are shown for one cycle of locomotion.

Finally, by viewing the entire network as an information channel, we can examine how well information is preserved through each layer of processing. This is best done using the information clamp assay, in which we consider a constant change in concentration *ċ* and compute *I*(*ċ*; *l*(*t*)) for the set of elements in layer *l* assuming a uniform distribution over *ċ*. Of course, the ASE layer has perfect information about *ċ* (gray, Fig 3). The largest loss of information occurs in the first interneuron layer, with AIY still preserving 80.2% of the available information in ASE (blue). Most of the information in AIY is preserved by the AIZ layer, which contains 76.3% of the original information (red). Due to the oscillating pattern generator input, information in the SMB layer fluctuates between 58.2% and 76.2% of the original information (orange), while the information preserved in the neck angle fluctuates between 0.08% and 58.6% (black). Averaged over a locomotion cycle, the neck angle contains about 48.4% of the original information. Functionally, the information in the neck guides behavior. Therefore, we can say that the system as a whole preserves about half of the information about *ċ* that was originally available from the sensors.

### Analysis by Neuron Class

Overall mutual information provides a broad foundation for understanding information flow in the network. In this section we analyze in more detail the information flow through each layer of the circuit. In particular, we study (a) the specific information that each neuron provides about particular changes in concentration; and (b) the pathways along which information is transferred from one neuron to another.

#### Information is Specialized in ASE

**Fig 4:**
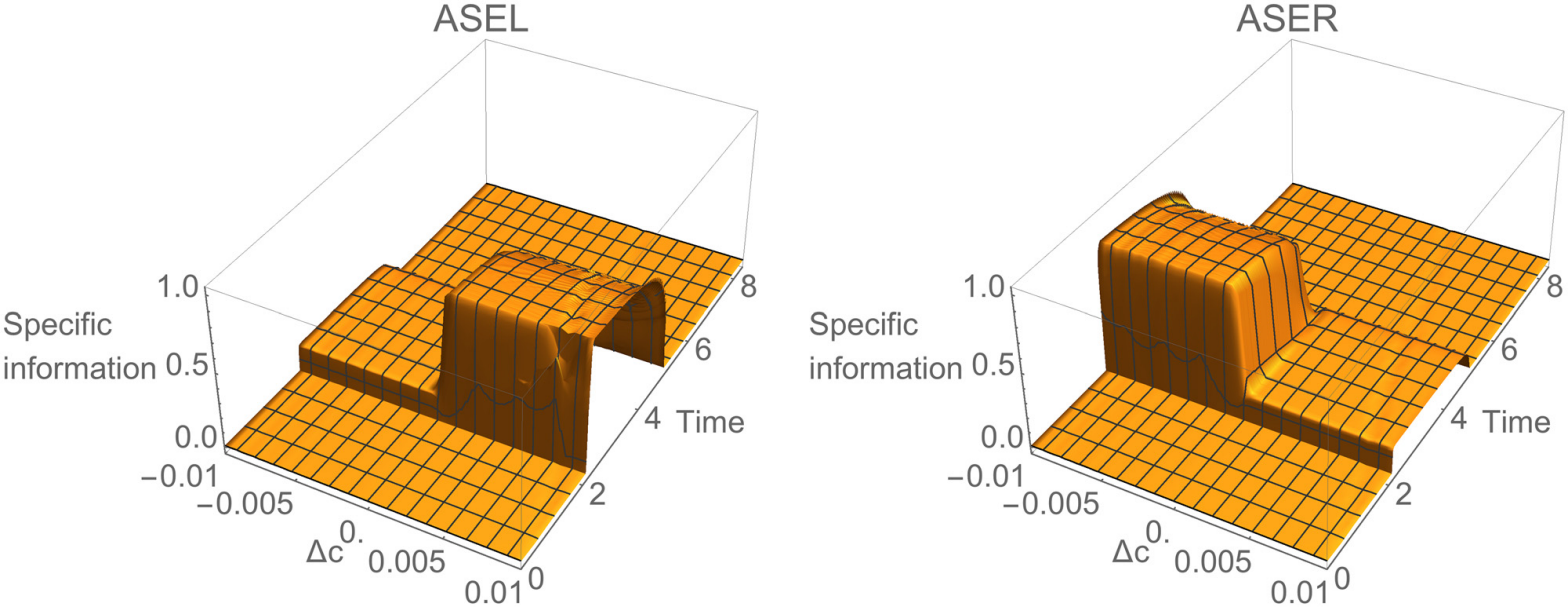
Information analysis for ASE during concentration step assay. Specific mutual information for left, *I*(Δ*c* = *k*;ASEL), and right, *I*(Δ*c* = *k*;ASER), cells over time.

We begin with the ASE chemosensory neurons. Since ASE responses are directly driven by changes in concentration, their informational analysis is straightforward. We use specific information to calculate the mutual information in ASE cells across different values for the change in concentration. Specifically, we compute the specific information that ASEL and ASER provide about Δ*c* as a function of the concentration step size *k*: *I*(Δ*c* = *k*; ASEL) and *I*(Δ*c* = *k*; ASER), respectively. Since ASEL and ASER detect only positive and negative concentration steps, respectively, it is not surprising that their specific information profiles reflect this pattern (Fig 4). Somewhat counterintuitively, each sensory neuron also provides a small amount of information about the stimulus range that it does not detect (smaller ridges in Fig 4). This is due to a kind of negative logic: for example, knowing that ASEL is off tell us that Δ*c* ≤ 0, which rules out half of the possible stimulus values and thus provides one bit of information about the stimulus. We call neurons such as these *informationally specialized* since they provide information about largely non-overlapping ranges of stimulus values.

#### Information is Asymmetric in AIY

**Fig 5:**
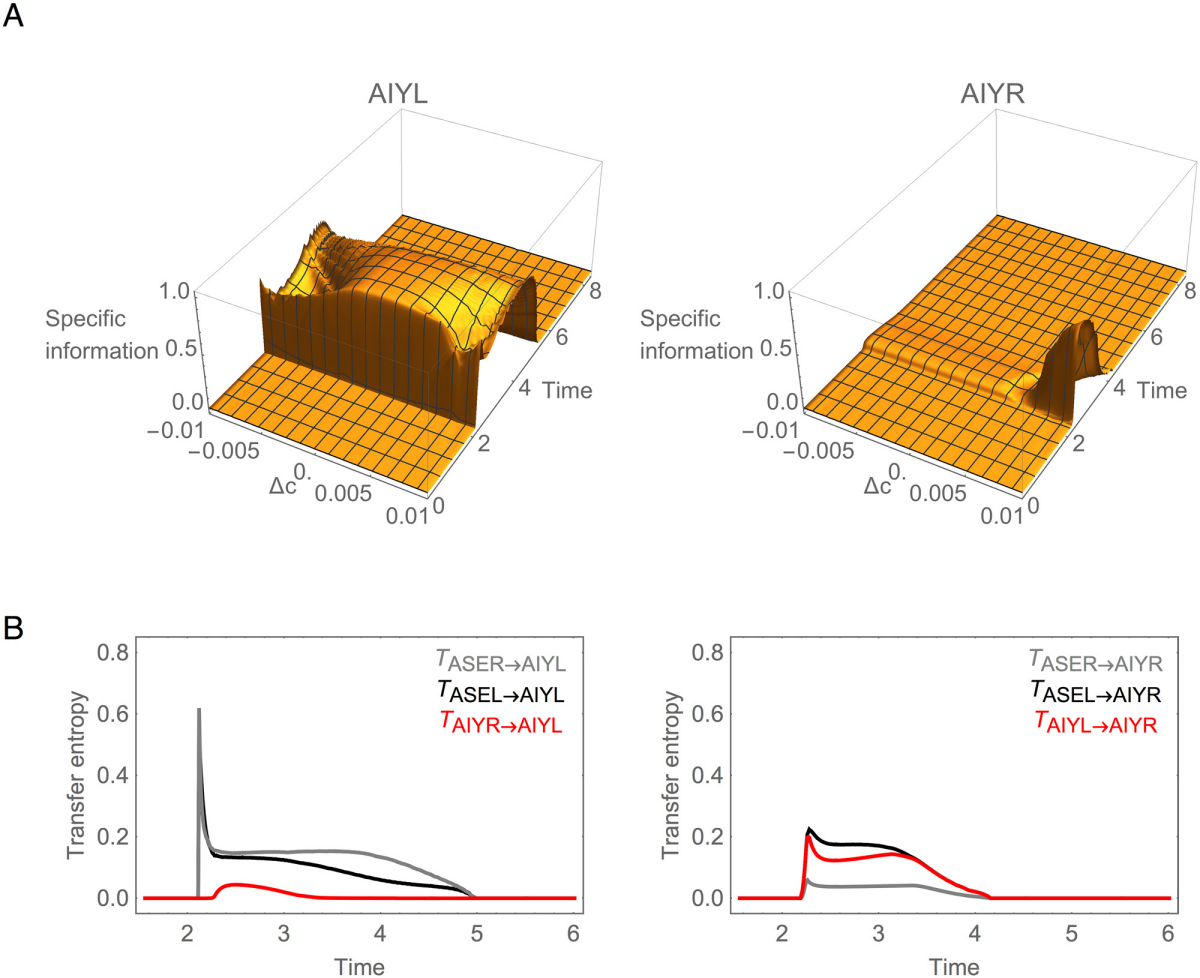
Information analysis for AIY during concentration step assay. (A) Specific mutual information for left, *I*(Δ*c* = *k*;AIYL), and right, *I*(Δ*c* = *k*;AIYR), cells over time. (B) Transfer entropy. Black and gray traces represent the transfer entropy to AIY through the chemical synapses from ASER and ASEL, respectively. Red traces represent the transfer entropy from the contralateral AIY cell through the gap junction.

Unlike the ASE sensory neurons, the AIY cells exhibit a strong asymmetry in the amount of information that each cell carries about changes in concentration. This asymmetry can be seen quite clearly in plots of specific information for AIY (Fig 5A). Although AIYL has more information about positive than negative steps, it maintains high information across the full range of Δ*c*. In contrast, AIYR contains information only about the largest positive steps (Fig 5A). We call neurons such as these *informationally asymmetric* since the information carried by one dominates the information carried by the other across the full range of stimulus values.

What is the origin of the informational asymmetry in the AIY layer? There are two possible pathways of information flow into the AIY cells: a direct route from ASE through the chemical synapses, and an indirect route from the other AIY cell through the gap junction between them (Fig 1). Using transfer entropy, we can quantify the contributions of these different pathways (Fig 5B). Overall, there is more information transferred into AIYL (left panel) than into AIYR (right panel). The AIYL neuron receives information from both ASE chemosensory neurons about positive and negative changes in concentration through the chemical synapses. The AIYR neuron, however, receives information from only one of the chemosensory neurons, ASER. And although there is information transferred from AIYL to AIYR via the electrical connection, the transfer is not sufficient to close the informational gap between the two cells. To confirm this, we measured the mutual information when blocking the AIY gap junction and observed no change in the informational asymmetry (S1 Fig).

What explains the observed informational asymmetry in AIY? The saturating nonlinearity of the synaptic transfer function (see Methods) constrains the cell to respond to changes in concentration within a certain range. This range is a function of each cell’s bias parameter in relation to the strength and sign of the incoming chemical synapses from the chemosensory neurons. Therefore, to understand the observed asymmetry between the two AIY cells, we have to analyze the parameters of the cells that determine their dynamical behavior (S2 Fig). Although the pair of incoming chemical synapses from ASEL and ASER are of similar strength and polarity for both AIY cells, the bias of the AIYR cell is far more negative than that of the AIYL cell, whose response range is nearly centered in the range of possible net input. It is this difference in the intrinsic properties of the AIY cells that creates the informational asymmetry.

#### Information is Symmetric in AIZ

**Fig 6:**
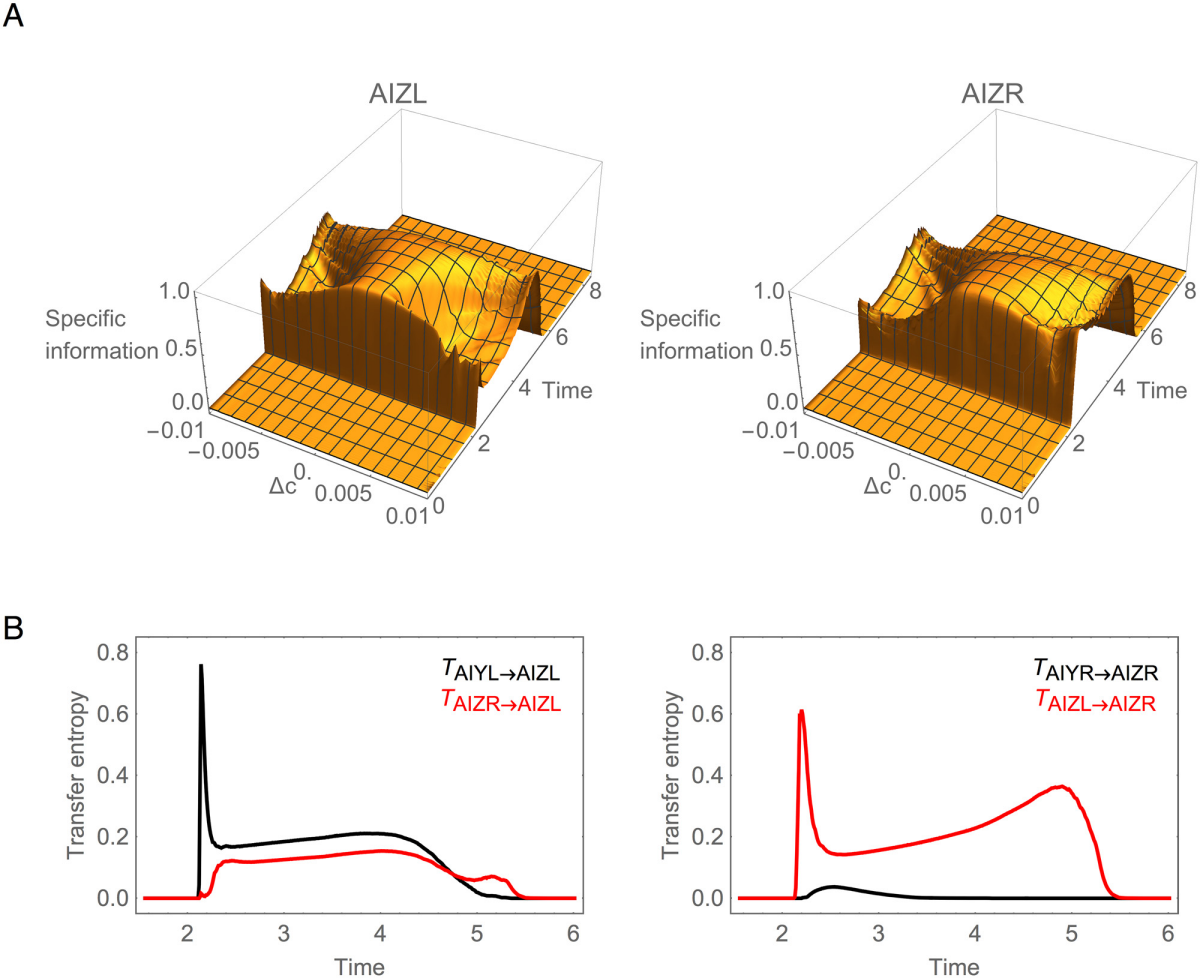
Information analysis for AIZ during concentration step assay. (A) Specific mutual information for left, *I*(Δ*c* = *k*;AIZL), and right, *I*(Δ*c* = *k*;AIZR), cells over time. (B) Transfer entropy. Black trace represents the transfer of information from AIY to AIZ through the chemical synapse. Red trace represents the transfer of information from the contralateral AIZ cell through the gap junction.

Despite the strong information asymmetry in the AIY layer, the information profiles become symmetric in the AIZ cells (Fig 6A). We call neurons such as these *informationally symmetric* since they each carry approximately the same amount of information across the full range of stimulus values.

What is the origin of this informational symmetry in the AIZ layer? There are again two pathways: a direct route through the chemical synapse and an indirect route through the gap junction (Fig 1). Using transfer entropy, we can quantify the contributions of these different pathways (Fig 6B). Immediately after the step occurs, AIZL receives the majority of information directly through the chemical synapse (black trace, left panel). In contrast, AIZR receives its information not through the chemical synapse (black trace, right panel), but rather through the gap junction (red trace, right panel). Thus, unlike in the AIY cells, the AIZ gap junction plays a major role in establishing the information profile in the AIZ layer. To confirm this, we measured the mutual information when blocking the AIZ gap junction and observed a substantial change in the information in AIZR that left the two cells informationally asymmetric (S1 Fig).

What explains the difference in information transfer between the AIY and the AIZ gap junctions? The gap junction connects two cells electrically so that current flows from the cell with more charge to the cell with less charge. As a result, the membrane potential of the two cells tend to equalize. Once equalized, any change in potential in one of the cells will cause a change in the potential of the other cell. Due to the nonlinearity of the synaptic transfer function, however, whether a change in potential will have an impact in the synaptic output (and thus downstream of the circuit) depends on where the potential is with respect to the sensitive region of the transfer function. This is determined by the cell’s bias parameter. Therefore, similarity of the bias parameter facilitates information exchange via a gap junction. In the case of AIY (S2 Fig), the difference in the bias of the left and right cells is such that any changes in potential in AIYR as a result from changes in potential in AIYL transmitted via the gap junction are lost due to the nonlinearity of AIYR’s synaptic transfer function. On the other hand, in the case of AIZ (S3 Fig), the similarity in the bias of the left and right cells is such that changes in the potential of AIZR as a result from changes in potential in AIZL transmitted via the gap junction results in effective changes to AIZR’s synaptic output.

#### Information Gating in SMB

**Fig 7:**
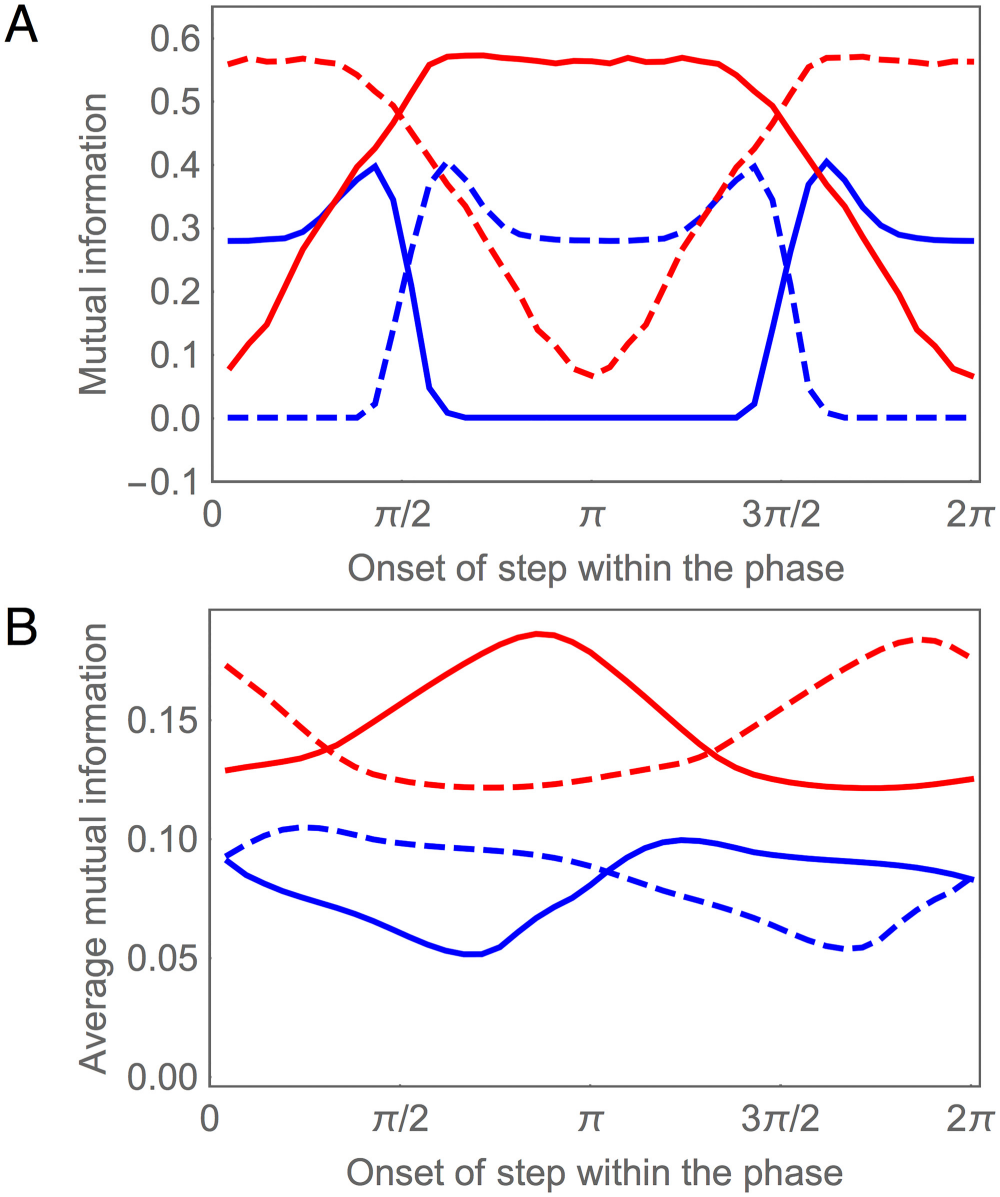
Information gating in SMB motor neurons during concentration step assay. (A) Mutual information for each SMB neuron as a function of the onset of the concentration step relative to the phase of locomotion. The mutual information is measured at a fixed delay of 50 msec after the step occurs. Left cells shown in blue. Right cells shown in red. Ventral (solid) and dorsal (dashed) traces. (B) Mutual information averaged over time as a function of the onset of the concentration step relative to the phase of locomotion.

Since the SMB neurons receive input from an oscillatory pattern generator in addition to the chemical synapses from the AIZ layer (Fig 1), their response to a concentration step Δ*c* depends on the phase *φ* of the oscillation when the step occurs. One way to visualize this phase dependence is to plot *I*(Δ*c*;SMB_*i*_) for each SMB neuron as a function of *φ* (Fig 7A). In order to simplify the plot, we show *I*(Δ*c*;SMB_*i*_) only at a fixed delay of 50 msec after a step occurs, which corresponds to the time it takes the information in the AIZ neurons to stabilize after a step (AIZ, Fig 2). Here we see that each SMB neuron acts as a kind of gate, allowing Δ*c* information from the AIZ layer to pass through at some phases, but blocking or strongly attenuating it at others. Of course, examining this effect at a single delay gives a very limited window into what is in fact a temporally-extended response. In order to visualize the cumulative effect of this response, we can average plots like Fig 7A over all possible delays for a full locomotion cycle (Fig 7B). Although the gating is seen less sharply in this case, the phase dependence of information transmission from the SMB layer is still quite clear.

Antiphase information gating in the dorsal and ventral motor neurons is responsible for the state-dependent response that allows the model worm to steer. A change in concentration received at different phases of locomotion produces identical chemosensory signals that are sent simultaneously to both the dorsal and ventral motor neurons, yet these signals have asymmetrical effects that allow the model worm to undergo either a dorsal or ventral turning bias, as appropriate for orienting correctly to the gradient. For example, if an increase in concentration received during a dorsal-to-ventral sweep attenuates dorsal turning, then the same increase in concentration received during a ventral-to-dorsal sweep should instead attenuate ventral turning. By opening and closing the flow of information through the dorsal and ventral motor neurons in antiphase, the worm generates different responses to the same stimuli depending on its phase of locomotion.

In order to understand how information gating is implemented at the neuronal level, we need to consider the synaptic transfer functions for the motor neurons (S4 Fig). From previous work [40, 43], we know that the sensitive region of the synaptic transfer functions of the motor neurons are shifted relative to the range of the oscillatory input. Consequently, when the dorsal motor neurons are in their sensitive region, the ventral motor neurons are not, and vice versa. It is this alternating pattern of saturating nonlinearity in the SMB cells that explains the information gating: while motor neurons on one side transfer information to the neck openly, motor neurons on the opposite side of the worm block the flow of information by saturating its output.

**Fig 8:**
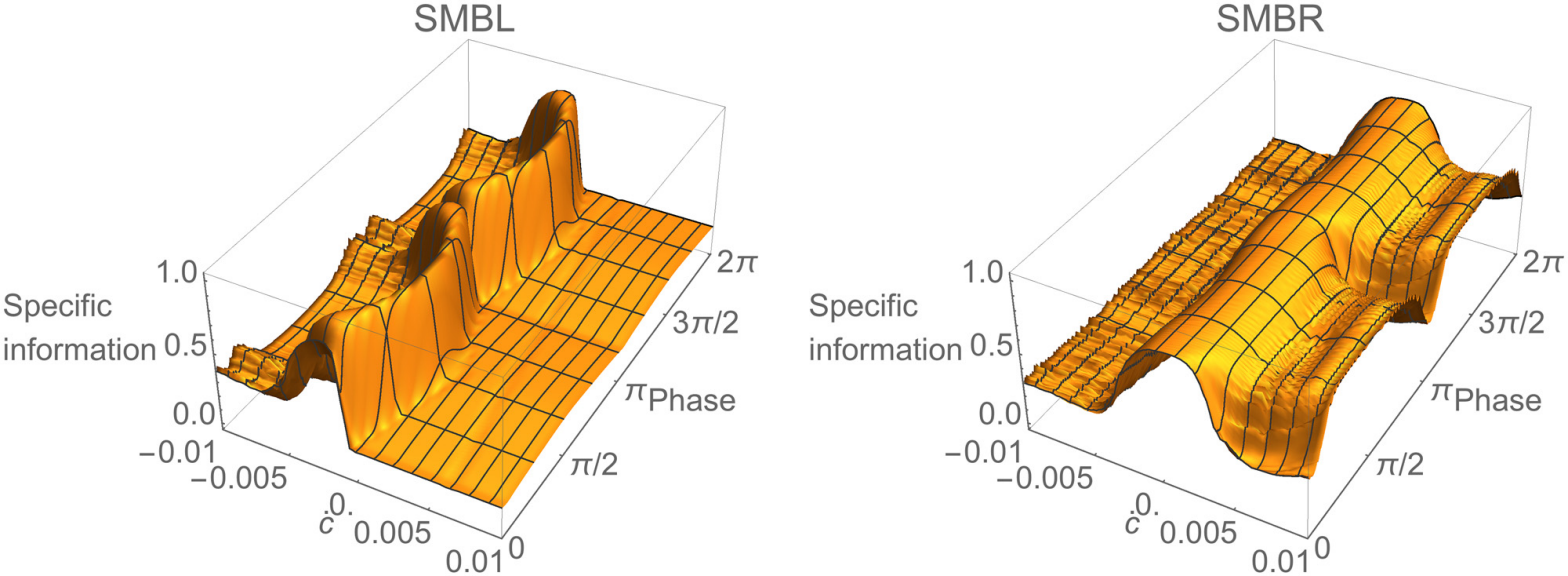
Information analysis in left and right pairs of SMB cells during information clamp assay. Specific mutual information for left, *I*(*ċ* = *k*;SMBL), and right, *I*(*ċ* = *k*;SMBR), pairs over time. Surfaces shown for one cycle of locomotion.

Finally, we examine how information about changes in concentration is distributed across the individual SMB cells. In order to more easily visualize the corresponding specific information, we make two simplifications. First, we switch to the information clamp assay in order to remove the phase dependence of the cell’s response to the concentration step assay. Second, we consider the dorsal/ventral pairs jointly on each side. This simplification is motivated by the fact that, because the network parameters are dorsal/ventral symmetric (see Methods), the dorsal/ventral information profiles on each side are identical except for a phase shift, whereas the left/right profiles are quite different (Fig 7B). The resulting plots of *I*(*ċ* = *k*;SMBL) and *I*(*ċ* = *k*;SMBR) are shown in Fig 8. Note that the *ċ* information in the right SMB motor neurons is strongly biased toward increases in concentration, whereas information in the left SMB motor neurons is strongly biased toward small decreases in concentration. However, despite this apparent specialization, SMBR carries more information than SMBL across the range of stimulus values. In the case of positive changes in concentration, SMBL carries no information, whereas SMBR carries some. In the case of negative changes in concentration, SMBR carries a similar amount of information as SMBL, so that a lot of the information about negative changes in SMBL is shared by SMBR. Thus, most of the information carried by the SMB layer resides in the SMBR pair, with the SMBL pair making only small contributions at very specific points in time.

### Functional Information in the Neck

The neck integrates information from the SMB motor neurons, converting it to a neck angle that steers physical movement. Like the motor neurons, the information profile of the neck also varies across the locomotion cycle due to the oscillation of the pattern generator. Using the information clamp assay, we examine how information about concentration changes in the neck angle varies with both time and stimulus, with particular interest in the behavioral significance of these variations.

**Fig 9:**
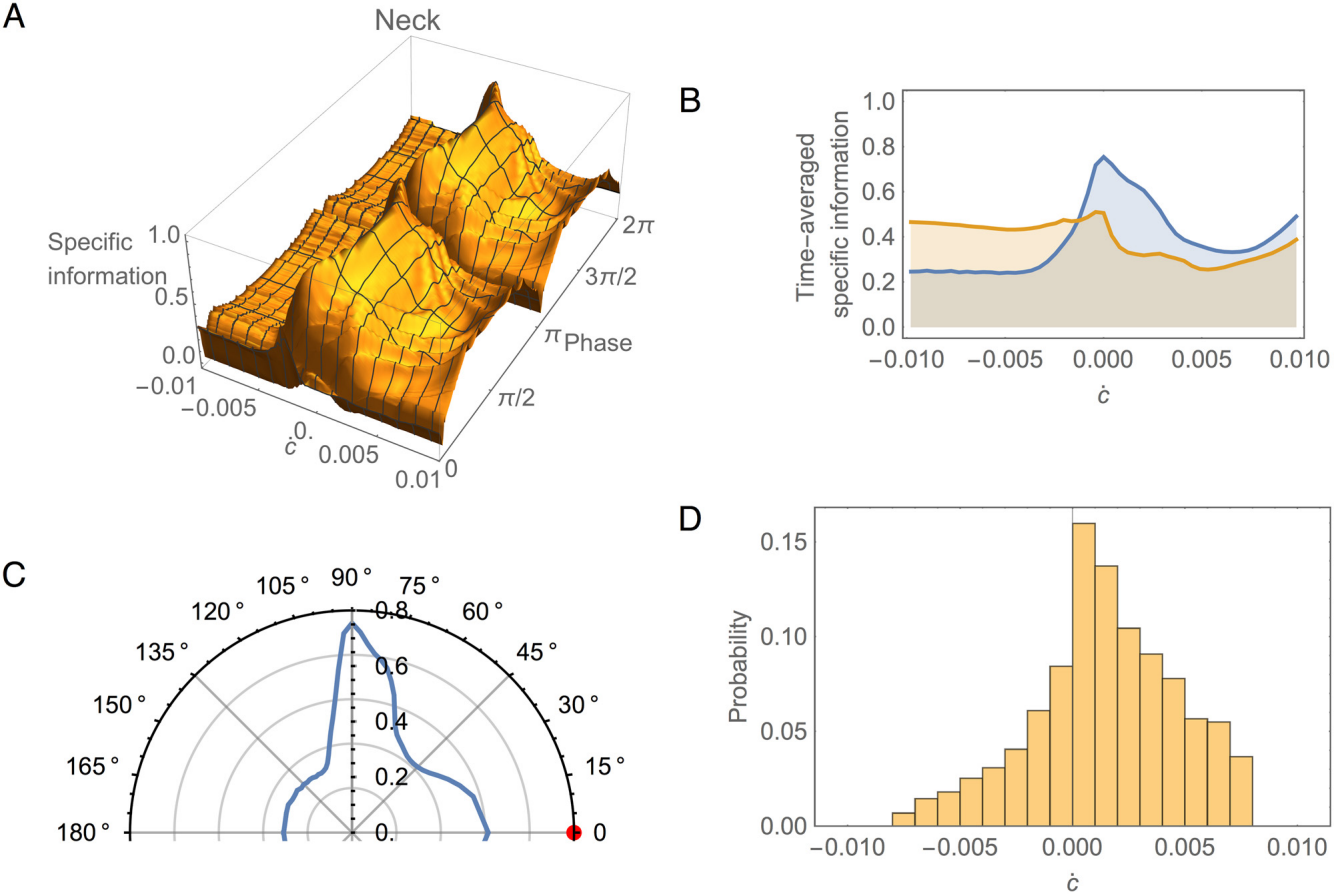
Information analysis in the neck during information clamp assay. (A) Specific information over time, *I*(*ċ* = *k*;*θ*). Surface shown for one cycle of locomotion. (B) Specific information averaged over time as a function of the change in concentration, calculated using a sample of the stimulus feature chosen from a uniform distribution (blue) and from the model-generated empirical distribution shown in panel D (yellow). (C) Specific information averaged over time as a function of the worm’s instantaneous orientation with respect to the implied peak of the gradient (red disk, 0 degrees). Data shown only from 0 to 180 degrees due to symmetry. (D) Empirical distribution of changes in concentration perceived by the simulated worm during a typical klinotaxis run.

What information does the neck carry about the stimulus in the environment? To answer this question we turn to the specific information in the neck, *I*(*ċ* = *k*;*θ*), as shown in Fig 9A. The first thing to note is that, despite the oscillatory information from SMB cells, the information in the neck angle remains remarkably consistent over time. This suggests the neck integrates successfully information from all motor neurons. The brief drop in information is due to the dorsal/ventral symmetry of the SMB cells. When the pattern generator crosses zero halfway through the locomotion cycle, the activity of dorsal and ventral SMB cells is the same, and therefore the neck has no information about which way to turn. Functionally this corresponds to the transition between ventral and dorsal bending in the neck. The second notable feature of specific information in the neck is its variation with *ċ*. Given the consistency of this information over time, the variation is best visualized by averaging over a locomotion cycle (blue trace, Fig 9B). Two properties immediately become clear from this plot. First, the neck has more information about small changes in concentration than about larger changes. Second, the neck carries more information about positive changes in concentration than about negative changes. In order to make sense of these results, we must place them within the context of the worm’s behavior.

In a freely moving worm, concentration changes result from the instantaneous movement of the worm’s head relative to the peak of the gradient. Fig 9C shows the time-averaged specific information in the neck as a function of the orientation of the head with respect to the direction towards the gradient peak (red disk). Note that the circuit receives the most information when the head is moving perpendicular to the direction of the gradient peak (90 degrees). Note also that the circuit has more information about concentration changes when the head is moving in the direction of the peak (0 degrees), than when moving away from it (180 degrees). This indicates that not all directions of movement are equally informative for the worm; movements perpendicular to the peak of the gradient, which generate only small changes in concentration, turn out to be the most informative.

Interestingly, the distribution of concentration changes encountered by a freely-moving worm engaging in klinotaxis behavior (Fig 9D) has a remarkably similar structure to the average specific information carried by the circuit (Fig 9B), with both having peaks at *ċ* ≈ 0 and a bias toward positive changes in concentration. In other words, the freely moving worm spends the majority of each run moving perpendicular or towards the gradient peak, and the information extracted by the klinotaxis circuit (Fig 9B) is well-tuned to the corresponding distribution of concentration changes encountered during klinotaxis behavior (Fig 9C).

In the absence of any experimental characterization of the actual distribution of concentration changes encountered by a worm performing klinotaxis, analyzing information flow under the assumption of a uniform distribution of sensory inputs is the best that one can do. However, we can use the distribution of concentration changes encountered by our model (Fig 9D) as an estimate of the corresponding distribution for the worm and then re-analyze the information flow using this predicted empirical distribution. When this is done, we observed that the specific information in the neck is more uniform across all stimulus values (yellow, Fig 9B), in contrast with the more highly skewed information with the uniform distribution (blue, Fig 9B). This seems consistent with the idea that the network has been optimized for the statistical structure of its environment. The specific information takes into account both how well a given stimulus is encoded and how “surprising” (i.e., improbable) it is, or equivalently, how much information we stand to gain by learning its value. Therefore, a more uniform distribution of specific information values with the empirical distribution suggests the neck carries more information about less surprising stimuli and less information about more surprising stimuli.

### Information architecture

**Fig 10:**
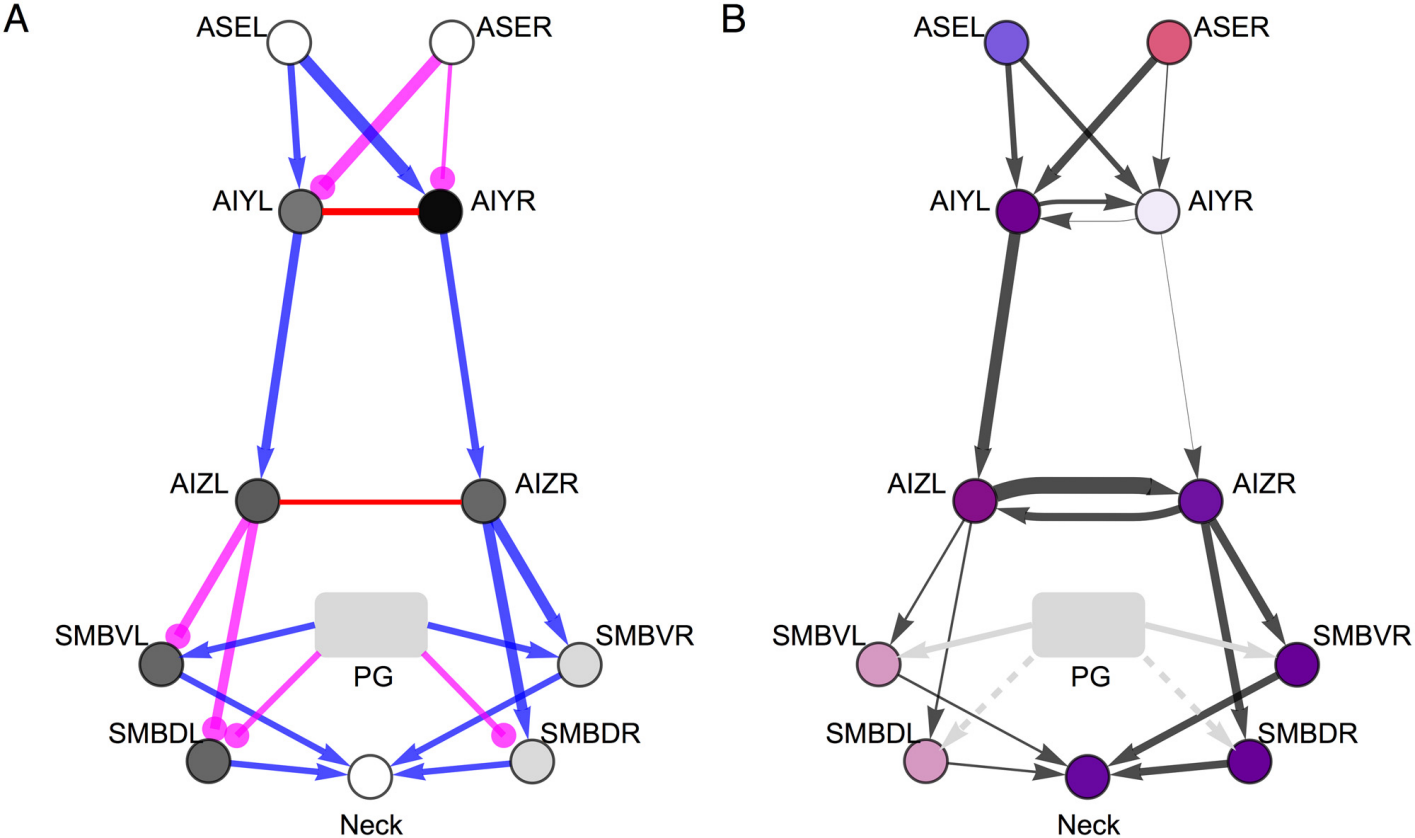
Structure and function in the best evolved circuit. (A) Structural architecture. The strength of the chemical and electrical connections in the circuit are represented by the thickness of the lines connecting the nodes. Excitatory chemical synapses are shown in blue. Inhibitory chemical connections are shown in magenta. Gap junctions are shown in red. Unlike chemical synapses, gap junctions are undirected. The bias of the cells are represented by the shade of gray of the node. The bias tunes the relationship between the cell’s membrane potential and it’s synaptic output, and is only shown for cells AIY, AIZ, and SMB (see Methods). (B) Information architecture. The average amount of information in each cell is represented by the opacity of the node. The type of specific information in each cell is represented by the color of the node, and was calculated using the time-averaged specific information. The proportion of blue/red indicates the amount of information the cell carries about positive/negative changes in concentration, respectively. The amount of information transferred between two cells is represented by the thickness of the arrows, and is calculated using the time-averaged transfer entropy. For ASE, AIY, and AIZ cells, we used the concentration step assay. For SMB cells and the neck, we used the information clamp assay.

Our detailed examination of the best circuit allows us to characterize its general pattern of information flow, which we will refer to as its *information architecture* (Fig 10B). The information architecture provides a static summary of the flow of information through the circuit. Several of the previously identified features are apparent from this diagram. First, we see that information is specialized in the two ASE cells. It is then transferred through the chemical synapses and combined in AIYL. AIYR receives little to no information through either of the chemical synapses or the gap junction. The information in AIYL is transferred through the chemical synapse to AIZL, and is then transferred through the gap junction to AIZR. Both AIZ cells transfer their information to the SMB cells downstream, although more is transferred from AIZR to SMBR. Finally, the SMB cells transfer information to the neck.

Interestingly, we can contrast the information architecture with the underlying parameters of the circuit (Fig 10A). For example, from the strengths of the incoming connections to AIY alone, we would expect information to be symmetrical in the AIY layer, rather than the observed asymmetry discussed previously within the context of the detailed analysis of AIY. Also, from the strengths of the gap junction, we would expect transfer to be stronger between the AIY cells than between the AIZ cells, rather than the other way around as discussed previously within the context of the detailed analysis of AIZ. Therefore, the information architecture of a circuit does not follow intuitively from the circuit’s structure. And in some cases, the information architecture can be rather different than the structural circuit. Ultimately, knowledge of the structural and information flow architectures of a circuit provide complementary insight into the operation of the circuit.

### Similar Information Architecture from Disparate Parameters

**Fig 11:**
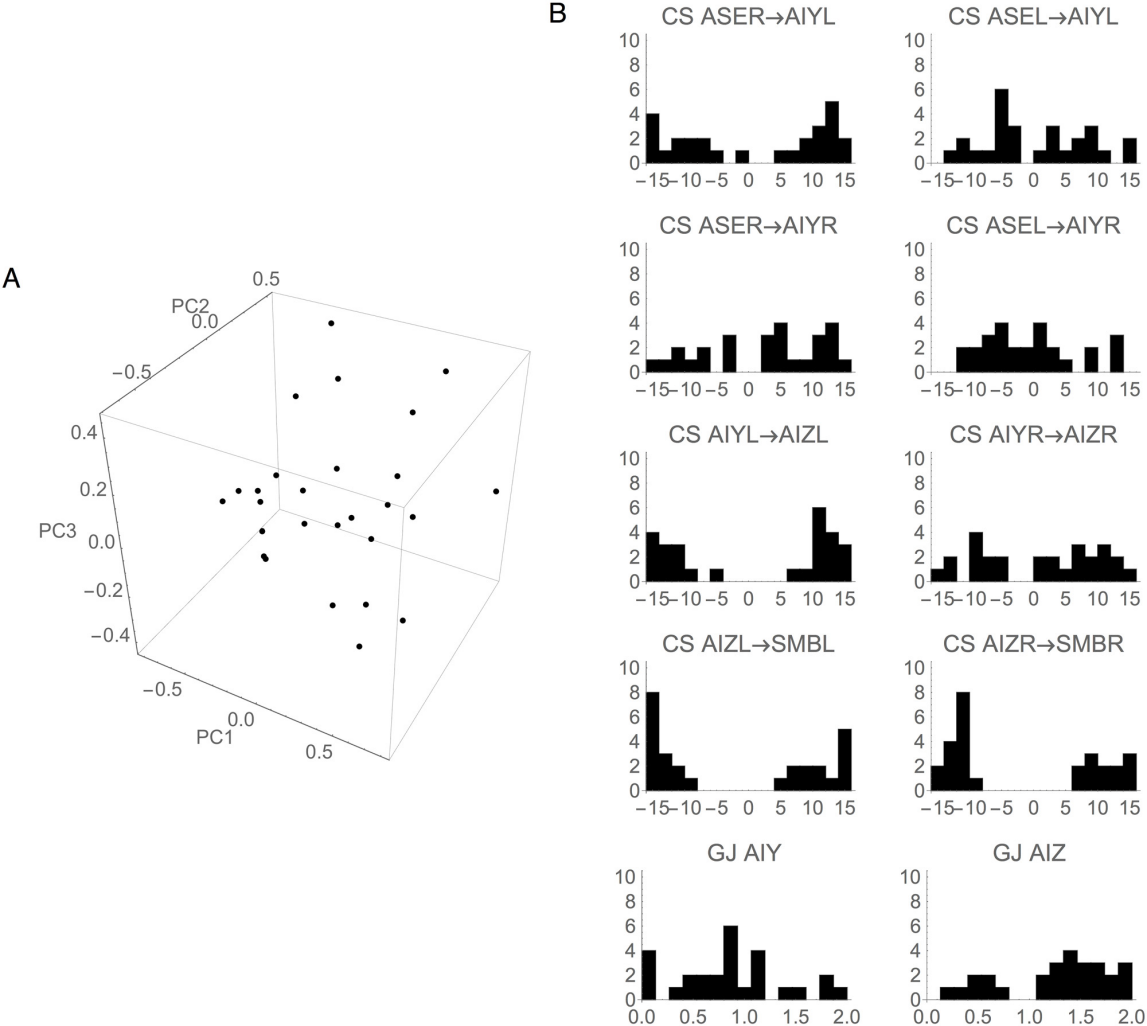
Cellular and synaptic properties of the ensemble of successful klinotaxis networks. (A) Principal component projection. The first three principal components capture 52.1% of the variance in the ensemble. There is no clear clustering of networks in this space. (B) Distribution of the chemical synapses (CS) and gap junctions (GJ) in the ensemble. Most connection strengths vary over the allowable ranges.

Up to this point, we have examined in some detail the information flow of the best circuit only. However, the complete ensemble of successful klinotaxis circuits contains many others with comparable performance [40]. Interestingly, the individual neuronal parameter values in the ensemble vary widely from circuit to circuit (Fig 11). In this section, we examine the extent to which the information architecture of the best circuit is representative of this neurophysiologically diverse set of successful circuits. We focus on the ensemble of highest-performers generated in previous work [40]. Specifically, we compare the information response profiles of the best circuit to the mean ± standard deviation response profiles of the ensemble.

**Fig 12:**
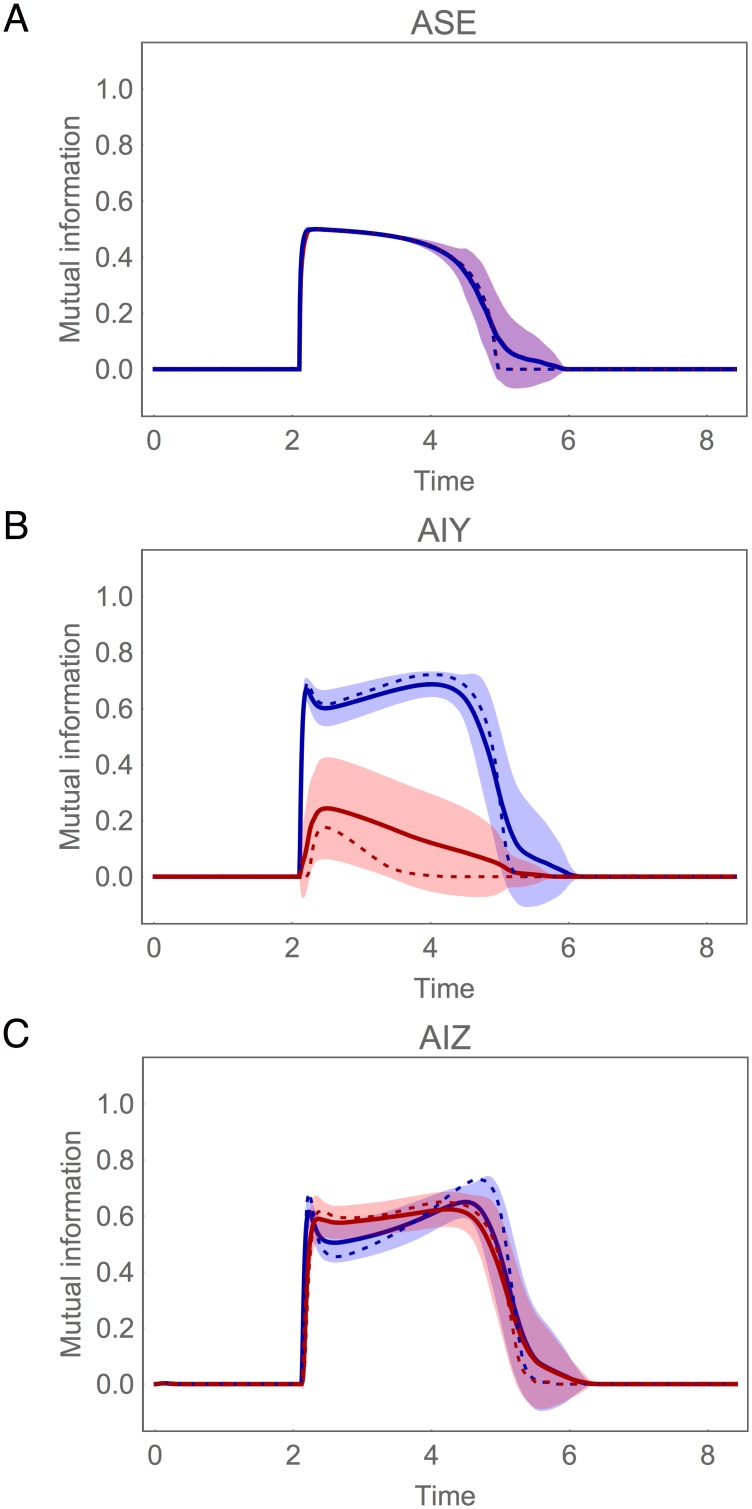
Mutual information during concentration step assay in the population of successful circuits. (A) Chemosensory neuron ASE: left (blue) and right (red) cell. (B) Interneuron AIY: cell with highest mutual information in blue, the other cell in red. (C) Interneuron AIZ: cell downstream from the AIY cell with highest mutual information in blue, the other cell in red. Mean (solid trace) and standard deviation (shaded area) for the ensemble of successful networks. Mutual information for the best circuit shown as a dotted trace.

The information profiles of the ASE, AIY and AIZ cells are shown in Fig 12 for the concentration step assay. The most important thing to notice about these plots is the strong similarity between the profiles of the ensemble mean (solid trace) and those of the best circuit (dotted trace). First, the rise in Δ*c* information in ASE is sharp and persists for a little over half a locomotion cycle (Fig 12A). Second, only one of the AIY cells integrates Δ*c* information, consistently creating a strong left/right information asymmetry in this layer of the circuit (Fig 12B). Finally, Δ*c* information is consistently symmetric across the AIZ cells (Fig 12C).

**Fig 13:**
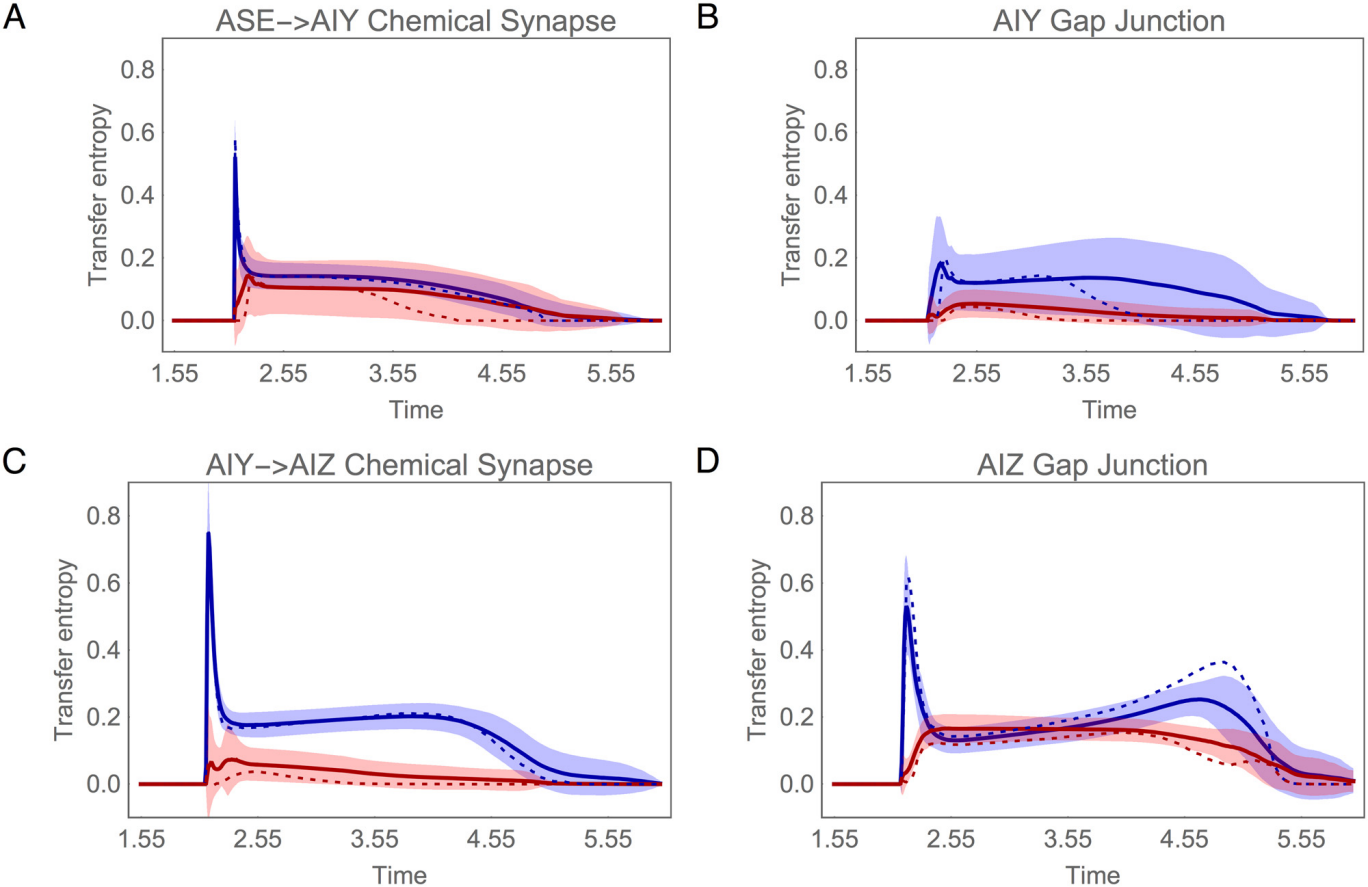
Transfer entropy in the population of successful circuits. (A) Through chemical synapses from ASE to: AIY cell with most information (blue), and to the AIY cell with lowest information (red). The traces show the mean (solid trace) and standard deviation (shaded area) for the chemical synapses from both ASE cells. (B) Through chemical synapse from the AIY cell with most information to the AIZ cell downstream (blue), and from the AIY cell with lowest information to the AIZ cell downstream (red). (C) Through the AIY gap junction: from the cell with most information to the cell with lowest information (blue), vice versa (red). (D) Through the AIZ gap junction: from the cell downstream of the AIY cell most information to the cell downstream of the AIY cell with lowest information (blue), vice versa (red). Mean (solid trace) and standard deviation (shaded area) for the ensemble of successful networks. Transfer entropy for the best circuit shown as a dotted trace.

The striking similarities between the best circuit and the ensemble average continue when we examine the pattern of information transfer between layers (Fig 13). In the AIY layer, the Δ*c* information is consistently transferred into the primary AIY cell via the chemical synapses from ASE (Fig 13A), with the gap junction playing a secondary roll (Fig 13B). In addition, in the AIZ layer, the Δ*c* information is first transferred from the primary AIY cell to the downstream AIZ cell via the chemical synapse (Fig 13C), and only then transferred via the gap junction to the other AIZ cell (Fig 13D). Thus, as in our analysis of the best circuit, we see that the gap junctions consistently play a major role in balancing information in the AIZ layer.

**Fig 14:**
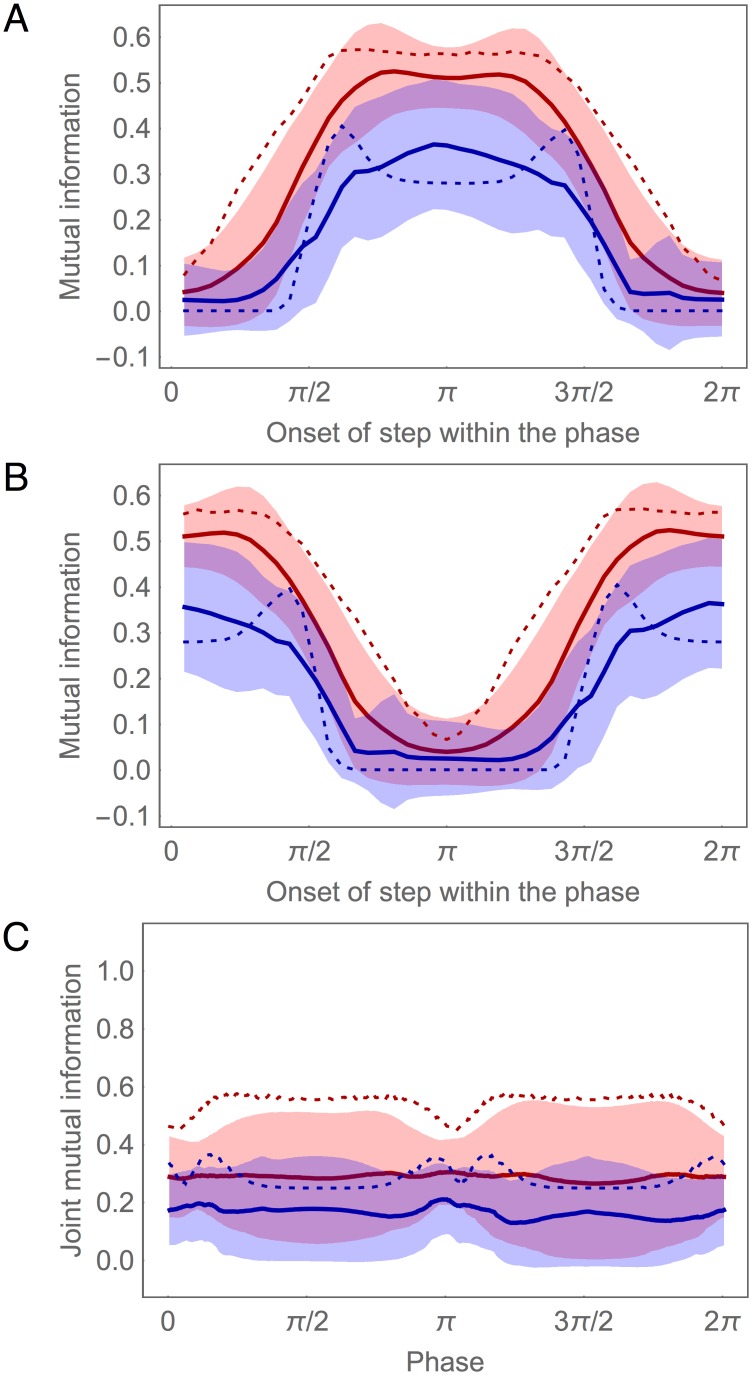
Information gating in the population of successful circuits. (A, B) Mutual information for each SMB neuron as a function of the phase of locomotion when the step in concentration is given, at a fixed delay of 50 msec after the step occurs. The four motor neurons are organized in two ways. The dorsal pair and ventral pair are categorized by their phase: Dorsal/ventral pairs with highest mutual information around *π* of the locomotion phase (A), pairs with highest mutual information around 0/2*π* (B). The left and right motor neurons are categorized by the cumulative information they carry: The cell with highest information is shown in blue, the other cell is shown in red. Mean (solid trace) and standard deviation (shaded area) for the ensemble of successful networks. (C) Mutual information for the joint left and right pairs during information clamp assay. Pair with highest mutual information shown in red, other pair shown in blue.

Despite greater variability in the information profiles for the SMB layer, there are still strong similarities between the best circuit and the ensemble average. For example, the concentration step assay reveals clear evidence of Δ*c* information gating in the SMB layer across the ensemble (Fig 14A and 14B). In addition, as with the best circuit, an analysis of mutual information for the information clamp assay demonstrates a left/right asymmetry in SMB across the ensemble, with one side consistently providing more information about *ċ* than the other (Fig 14C). However, we also see evidence that the best circuit is a bit of an outlier in the SMB layer: the ensemble has more balanced information in the left/right pairs than does the best circuit.

**Fig 15:**
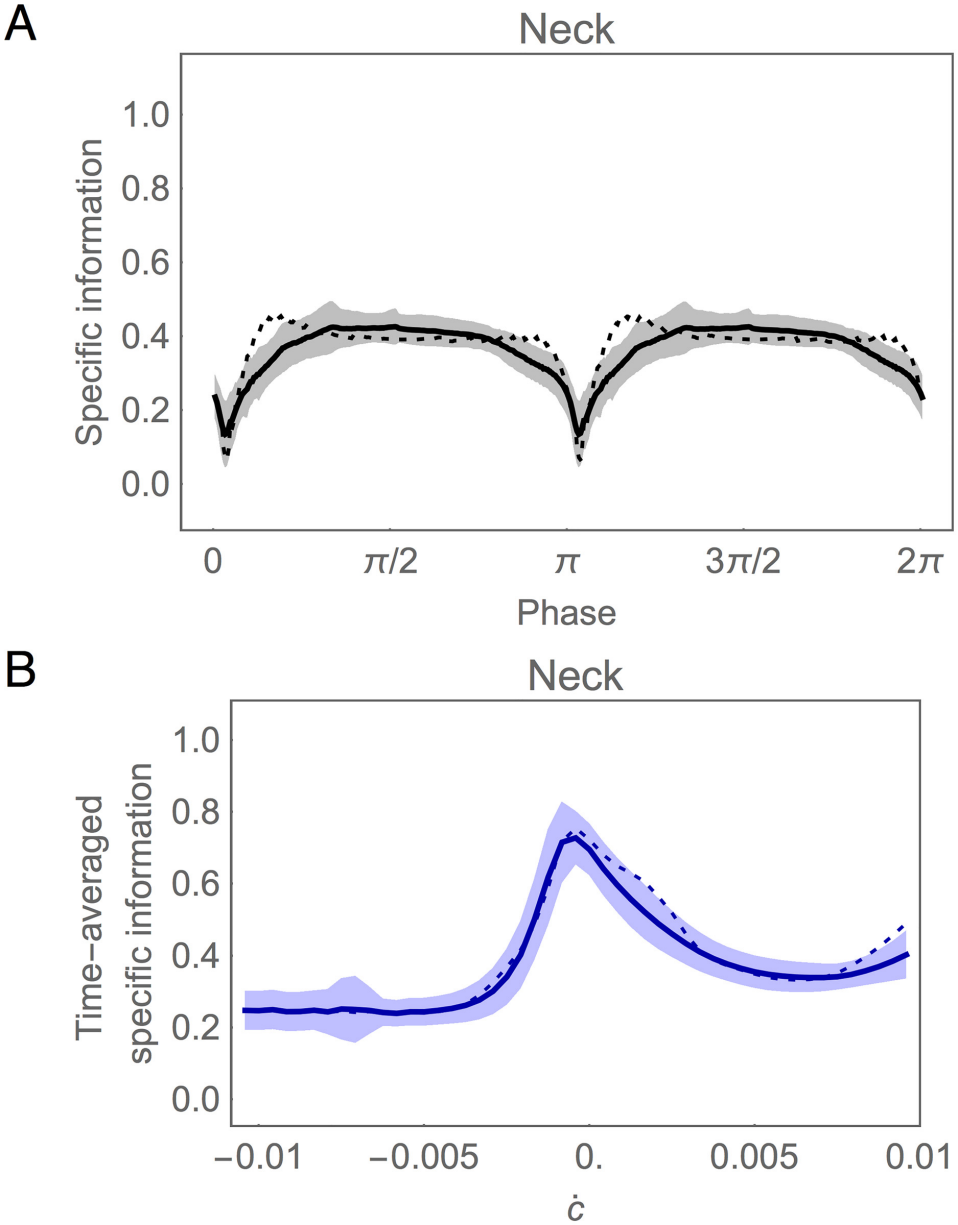
Functional information in the neck during information clamp assay in the population of successful circuits. (A) Mutual information over time shown for one cycle of locomotion. (B) Specific information averaged over time as a function of the change in concentration. Mean (solid trace) and standard deviation (shaded area) for the ensemble of successful networks. Best circuit (dotted trace).

Finally, we consider information about *ċ* in the neck using the information clamp assay (Fig 15). Despite the outlier status of the best circuit in the SMB layer, at the neck the ensemble mean information profiles are very close to those of the best circuit, with relatively small dispersion. Specifically, as in the best circuit, the mean amount of information about *ċ* across the ensemble remains relatively constant throughout the locomotion cycle, preserving about half of the original information available from the chemosensors (Fig 15A). In addition, the mean amount of specific information about particular values of *ċ* across the ensemble, averaging over one locomotion cycle, is peaked at *ċ* ≈ 0 and higher for increases in concentration than for decreases, as in the best circuit (Fig 15B).

We conclude from this analysis that the results obtained from the best klinotaxis circuit are in fact fairly representative of the entire ensemble of successful circuits (see Fig 10B). This is a somewhat surprising conclusion, given the large variations in synaptic strengths, intrinsic neuronal properties (Fig 11), and circuit dynamics across the ensemble [43]. The consistency of the information flow architecture across the ensemble suggests that information flow analysis may be particularly well-suited for capturing general principles of operation that cut across the substantial variability and complex idiosyncrasies of individual klinotaxis circuits.

## Discussion

In this paper, we set out to analyze how information about changes in salt concentration flows through a putative minimal circuit for *C. elegans* klinotaxis [40, 65]. Our goal was to demonstrate how the tools of information theory can be used to characterize the flow of information throughout a complete sensorimotor circuit: from stimulus, to sensory neurons, to interneurons, to motor neurons, to muscles, to motion. We proceeded in three stages. First, we examined the overall flow of information by calculating mutual information across time for each component of the best circuit. Second, we analyzed in detail each layer of this circuit. We considered information across stimulus values for each cell, and we quantified information transfer between individual circuit elements. Finally, we considered the similarities and differences between the best klinotaxis circuit and the rest of the model ensemble.

Information flow analysis gave insight into specific questions about the neural basis of salt klinotaxis in *C. elegans*, centered around two issues that make this behavior particularly interesting. First, given the functional asymmetry of the ASE chemosensory cells [42], the klinotaxis circuit has to integrate information about positive and negative changes in concentration. How is this integration achieved? Second, unlike klinokinesis [66], the other chemotaxis strategy that the worm employs, klinotaxis requires state-dependence: the network has to combine information from the environment with its own internal state to produce an adequate response [43]. How does this integration occur?

Given that information about positive and negative changes in concentration is segregated in the chemosensory neurons (ASEL detects upsteps in salt concentration and ASER primarily detects downsteps in salt concentration [42]), the klinotaxis circuit must ultimately integrate this information in order to steer. In principle, integration could occur in any layer of the minimal circuit. However, given that each AIY cell receives connections from both ASE chemosensors [31], the AIY layer is in the most favorable position. There are several different ways that integration could be achieved in the AIY layer: (1) Both AIY cells could integrate concentration information via the chemical synapses from ASE; (2) One AIY cell could integrate the information via its chemical synapses and then pass it to the other via the gap junction between them; (3) Only one AIY cell could integrate the concentration information. Of these three possibilities, option (3) requires the fewest parameters to be tuned and it is this option that we consistently found in our model ensemble. Thus, our analysis makes a specific prediction about the information profile in AIY. Given the ubiquity of this profile in the ensemble, a failure of this prediction would suggest that the assumptions behind the minimal model need to be revisited.

Strictly speaking, the integration of ASE information is only necessary if we assume that access to high amounts of information about the entire range of concentration changes is required for successful klinotaxis. Our analysis showed that, although each sensory neuron provides perfect information about only half of the range of concentration changes, each sensory neuron also provides a small amount of information (1 bit) about the other half of the range. This suggests that either chemosensory neuron alone could in principle drive at least a rudimentary form of the behavior if only a small amount of information about the entire range of concentration changes is required. For example, an ASEL-only circuit could only make graded adjustments when it was moving toward the peak, but it could still detect when it was moving away from the peak via the lack of activity in ASEL. Indeed, ablation studies have shown that an ASER-only circuit can successfully chemotax, but the results have been mixed for ASEL-only circuits [41, 65]. Simulated ablation studies in a previous model have also demonstrated successful chemotaxis behavior for ASEL-only and ASER-only conditions [43]. However, in both experiments and simulations, the single-ASE behavior is quite different from normal klinotaxis and the success rate varies significantly with environmental circumstances.

Our analysis also provides insight into the mechanism by which the klinotaxis circuit combines information about concentration changes with information about the phase of its locomotion to steer in the right direction at the right time. We know from previous studies that the klinotaxis circuit must be state-dependent: the correct response to a given stimulus depends on the phase of the head swing when it is received [43]. For example, an upstep received during a dorsal-to-ventral swing should produce an increase in the ensuing dorsal turn, whereas an upstep received during a ventral-to-dorsal swing should produce a decrease in the same turn. Our previous analysis suggested how saturating nonlinearity in the SMB cells might account for this state-dependence [40]. Here we place this mechanism in a broader context as a kind of information gate, with the circuit using the oscillatory signal to alternately open and close the flow of concentration information through the motor neurons. Since this oscillatory signal is antiphase for dorsal and ventral pairs of motor neurons, the timing of the gates is also antiphase. This leads to different responses to the same upstep or downstep stimuli at any specific time, depending on which of the gates are open and which are closed. Although the information gating mechanism is crucial for the state-dependence in klinotaxis, the proposal that it occurs in the SMB neck motor neurons is a result of the model’s assumption that the neck motor neurons receive antiphase oscillatory input from the worm’s locomotion. In principle, the gating can occur in any component of the network that receives the anti phase oscillatory input generated during locomotion, including in the interneurons or even in the head and neck muscles themselves.

In addition to specific questions relating to the neural basis of *C. elegans* salt klinotaxis, access to an ensemble of models whose parameters are fully known, and from which we can easily generate time-series recordings under any condition, allowed us to explore the relationship between the information flow of a circuit and the mechanistic understanding that we can derive from knowledge of its parameters. Information flow analysis provided insight into crucial aspects of the operation of the circuit, albeit at a relatively high level of abstraction: AIY cells were informationally asymmetric, AIZ cells were informationally symmetric, and SMB cells acted as information gates in phase with the worm’s locomotion. In order to answer questions about the mechanistic underpinnings of these features, we had to consider the specific parameters and the dynamics of the model. The suggestion is that the higher level of abstraction involved in information flow analysis can be useful to focus more detailed analysis of the underlying parameters and dynamics of the model on only the most functionally relevant parts. This is a particularly relevant point given that technology for generating time-series recordings from intact biological systems during behavior is growing faster than the characterization of the underlying biophysical properties of their relevant circuits.

Even if we had all of the details about the nervous system of a biological organism, understanding the principles underlying the neural basis of its behavior remains one of the main challenges in neuroscience. How do we combine the diverse and large amount of data needed to arrive at a set of principles for how the organism generates behavior from the dynamical interaction between its brain, its body and its environment? By focusing on a higher level of abstraction, information flow analysis captures unique insights into the operation of the circuit in two interesting ways. First, it allows the analysis to focus on the functionally relevant aspects of the circuit. Second, it allows for a more manageable comparison between circuits that perform the same behavior, even if their biophysical properties are entirely different. This was particularly useful in the ensemble of klinotaxis circuits, where despite large variations in individual neurophysiological parameters, the analysis revealed a single information flow architecture that was unique among all high-performing circuits in the ensemble. Indeed, the uniqueness of the klinotaxis information architecture suggests an interesting general lesson for neuroscience: Perhaps the truly universal principles are to be found not among the neurophysiological details, which can vary substantially [52–54], but instead at the more abstract level of patterns of information flow within a nervous system.

Despite being underdetermined by the available biological data, the uniqueness of the information flow architecture among the ensemble of circuits suggests that the constraints on which the model are based are reasonably strong. It would be very satisfying if the information flow architecture that we have identified turns out to be the one utilized by the worm. However, this architecture is only as good as the assumptions that went into the model. It is conceivable that, as the model assumptions are revised in light of new experimental data, key features of the information architecture will change. We mention briefly a few different directions for expanding the model. First, there are several candidate neurons that would be particularly useful to include in next iterations of the model, including chemosensory neurons ADF and ASH [62, 65] and interneuron classes AIA and AIB [63], all of which have direct connections with one or more neurons in the minimal circuit [31]. Second, the specific location of the postulated information gating depends on which components of the circuit receive the oscillatory input. Third, as we learn more about the neurophysiology of the underlying circuit, it may also become necessary to complicate the neural model that we currently employ. Recent advances in optogenetics in the freely-moving worm [67], as well as new experimental designs to study head swings in microfluidic devices [68] will accelerate the characterization of the neurophysiology in the proposed circuit. Finally, we have recently demonstrated that the minimal klinotaxis circuit is sufficient to steer a more realistic neuromechanical model of the nematode’s full body [69]. Understanding the flow of information in such a model will allow us to make predictions that are more easily testable in the worm.

The information analysis used to understand the klinotaxis circuit in this work is general enough to be applied to any brain-body-environment system. Therefore, an important direction for future work is to continue to develop the tools of information dynamics. We mention briefly three directions. First, when analyzing the information in a system, it is necessary to consider not only the information carried by individual variables, but also the information that may be encoded redundantly or synergistically by multiple variables. Although the concepts of synergy and redundancy have been of great recent interest in several areas in neuroscience [70, 71], the standard measures confound synergistic and redundant interactions and have problematic interpretations when more than three variables are involved [72–75]. We, along with several other groups, are currently working to develop measures of synergy and redundancy that would overcome these problems [25, 70, 71, 76, 77]. Second, in this paper we studied information flow only under open-loop conditions, meaning that the worm’s movement did not influence the concentration changes that it experienced, which were assumed to be uniformly distributed throughout. However, the worm’s movements obviously influence the statistical properties of its perceived concentration changes, which can be thought of as a specific instance of a general phenomenon known as “information self-structuring,” where an organism actively selects and shapes the sensory inputs that it receives through its actions [78]. An important direction for future work is to analyze the information flow of a sensorimotor circuit when it is in closed-loop interaction with its environment. Finally, to perform our analysis we had access to unlimited, noiseless data. Applying a similar analysis to cell recordings presents a number of challenges with respect to the limits of resolution and noise (for recent work applying information measures to experimental data, see [28, 79–82]).

## Supporting Information

S1 Fig Effect of blocking the gap junction on the information profiles of interneurons AIY and AIZ. The solid traces depict the mutual information of left (blue) and right (red) cells over time in the circuit. The dashed traces depict the mutual information when the gap junction has been blocked. (A) Blocking the AIY gap junction does not affect the informational asymmetry in AIY. (B) Blocking the AIZ gap junction affects the amount of information in AIZR, disrupting the overall information symmetry in AIZ.
(TIF)

S2 Fig Mechanism of information asymmetry in AIY. Synaptic transfer functions for the left (A) and right (B) AIY cells (solid black). Resting potential of the cells shown with dashed black line. ASER connects to both AIY cells through an inhibitory chemical synapse. ASEL connects to both AIY cells through an excitatory chemical synapse. Activity in ASER/ASEL drives the inputs to both AIY cells into the red/blue region, respectively. The relative strength of the connections is shown by the size of the region. The responsiveness of AIYL is the result of the alignment between the sensitive area of the synaptic transfer function and the range of possible net input. The bias in AIYR shifts the synaptic transfer function, leaving the cell sensitive only to the largest positive changes in concentration. Changes in the membrane potential in AIYL transmitted through the gap junction are equally ineffective to AIYR due to the shifted sensitive region.
(TIF)

S3 Fig Mechanism of information symmetry in AIZ. Synaptic transfer functions for the left (A) and right (B) AIZ cells (solid black). Resting potential of the cells before effects from the gap junction shown with dashed black line. Resting potential of the cells after equalization from the gap junction exchange shown with red dashed line. AIZ cells have incoming excitatory chemical synapses from AIY cells, left and right respectively. Therefore, activity in AIYR/AIYL drives the inputs to AIZR/AIZL into the gray region, respectively. The relative strength of the connections are shown by the size of the region. However, because AIYR shows very little activity, changes in membrane potential in AIZR are not due to the chemical synapse; instead they are due to changes in the membrane potential of AIZL through the gap junction. Unlike in AIY, the balance of the resting potential and the sensitive region of the synaptic transfer function in both AIZ cells results in a response to changes in concentration to negative and positive changes in concentration.
(TIF)

S4 Fig Mechanism of information gating in SMB. Synaptic transfer functions for the left (A) and right (B) pair of dorsal and ventral motor neurons, SMBL and SMBR, respectively (black trace). Instantaneous synaptic output as a function of net input when the head sweep oscillation is present (brown trace). Shaded areas show the range of oscillation due to the incoming connections from the pattern generator. For each of the SMB pairs, the input to the dorsal and ventral cells moves out of phase over the brown trajectory. As a result, when the dorsal motor neuron is at point *a* in the curve, the ventral motor neuron is at point *b*, and vice versa. Red and blue arrows show the effects of negative and positive changes in concentration on the input to the motor neurons, respectively. Due to the shift in the biases for the synaptic transfer functions, a change in concentration sometimes results in a change in the dorsal but not the ventral synaptic output, and viceversa. For the SMBL pair (A), a change in concentration results in a change in the synaptic output of the neuron in *b*, but not of the neuron in *a*. For the dorsal/ventral SMBR pair (B), *a* and *b* represent the opposite regions: the neuron at *a* is more sensitive to changes in input than the other neuron at *d*. To different degrees, the same is the case for other points along the curve. In both pairs of SMB neurons, the result is an antiphase dorsal/ventral gating mechanism.
(TIF)
