# Supplementary figures and images for: Information Flow through a Model of the C. elegans Klinotaxis Circuit
Source: PLoS One. 2015 Oct 14;10(10):e0140397. doi: 10.1371/journal.pone.0140397 (PMC4605772; doi:10.1371/journal.pone.0140397)

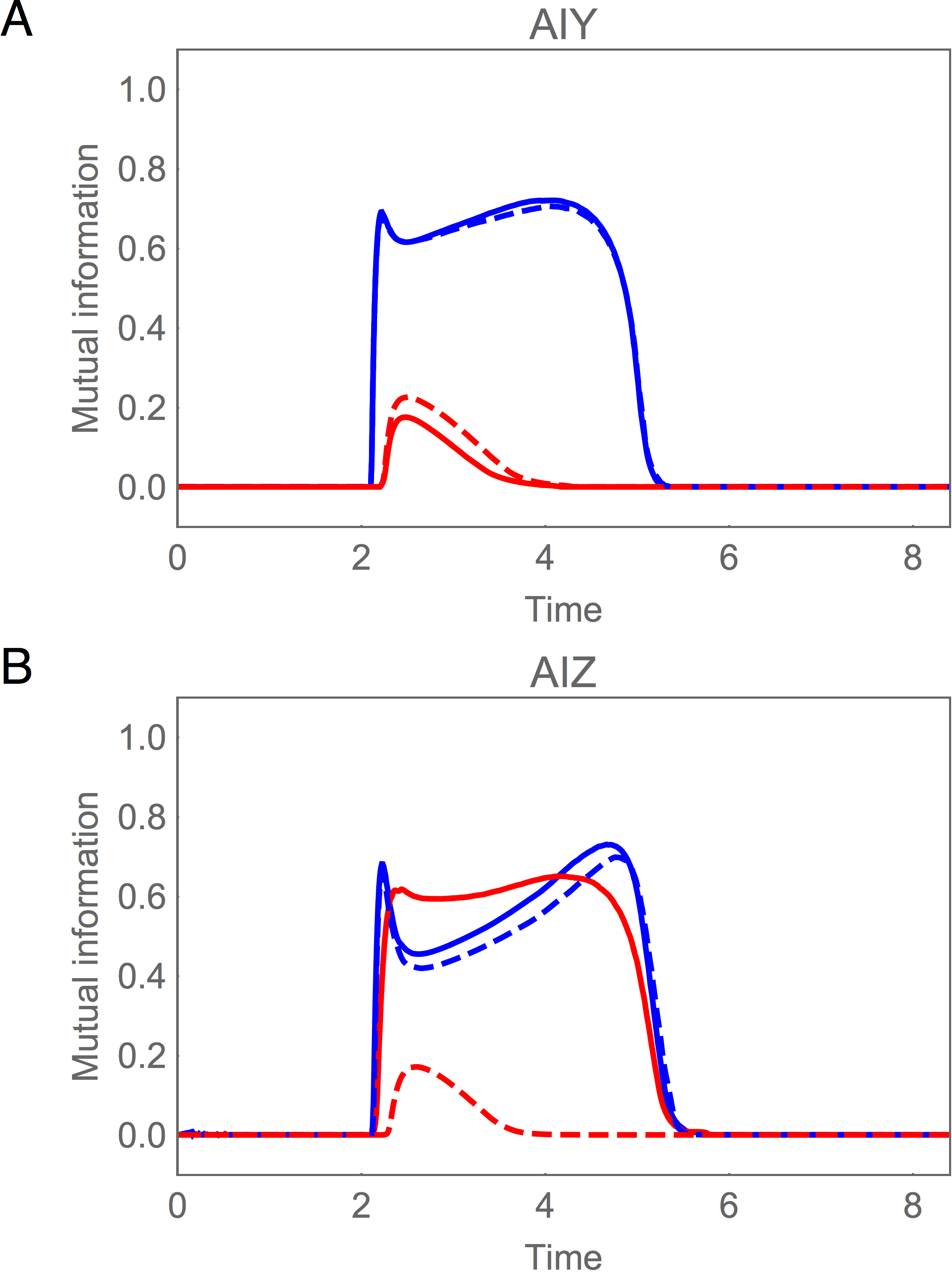

Supplement: S1 Fig — The solid traces depict the mutual information of left (blue) and right (red) cells over time in the circuit. The dashed traces depict the mutual information when the gap junction has been blocked. (A) Blocking the AIY gap junction does not affect the informational asymmetry in AIY. (B) Blocking the AIZ gap junction affects the amount of information in AIZR, disrupting the overall information symmetry in AIZ. (TIF) [file pone.0140397.s001.tif]

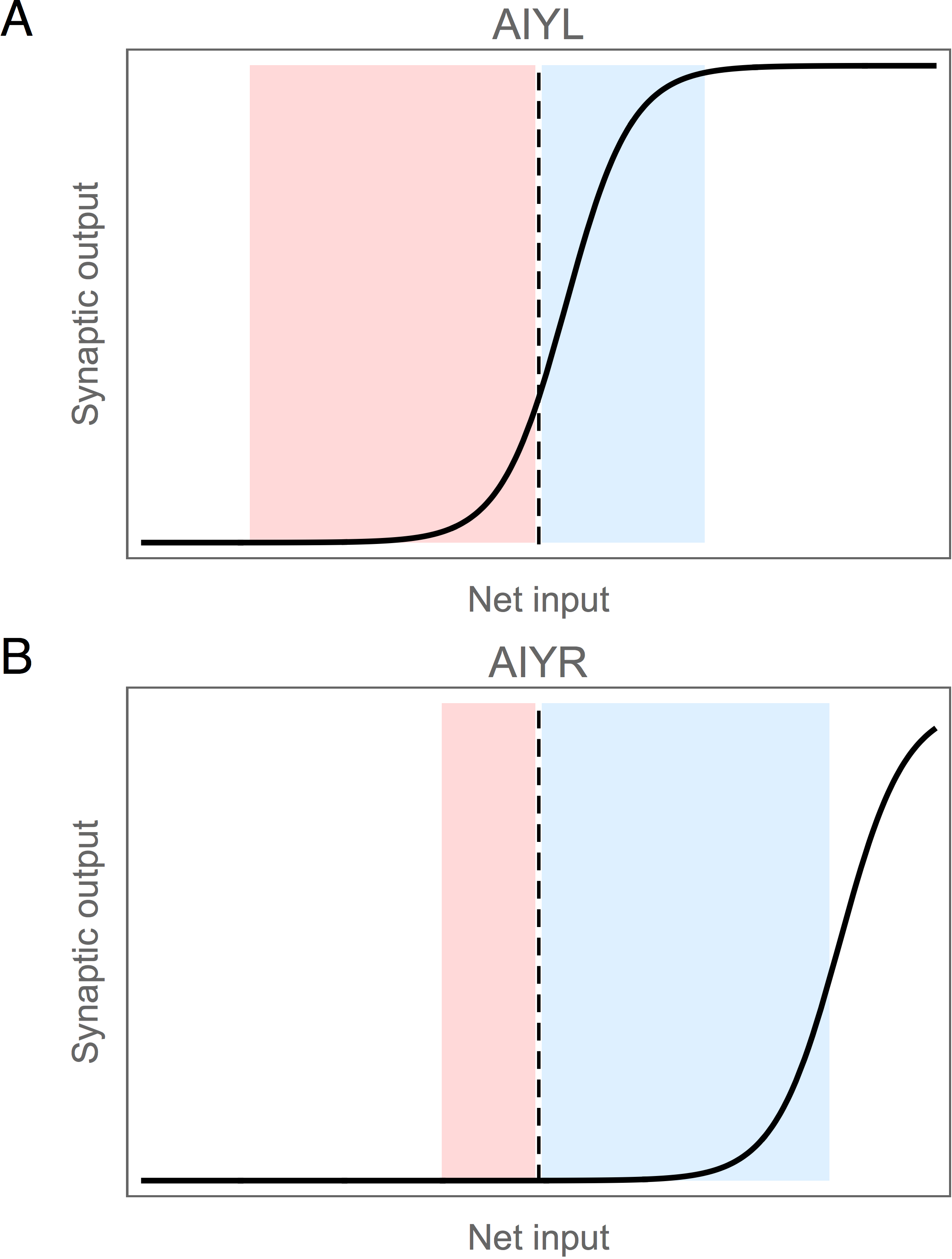

Supplement: S2 Fig — Synaptic transfer functions for the left (A) and right (B) AIY cells (solid black). Resting potential of the cells shown with dashed black line. ASER connects to both AIY cells through an inhibitory chemical synapse. ASEL connects to both AIY cells through an excitatory chemical synapse. Activity in ASER/ASEL drives the inputs to both AIY cells into the red/blue region, respectively. The relative strength of the connections is shown by the size of the region. The responsiveness of AIYL is the result of the alignment between the sensitive area of the synaptic transfer function and the range of possible net input. The bias in AIYR shifts the synaptic transfer function, leaving the cell sensitive only to the largest positive changes in concentration. Changes in the membrane potential in AIYL transmitted through the gap junction are equally ineffective to AIYR due to the shifted sensitive region. (TIF) [file pone.0140397.s002.tif]

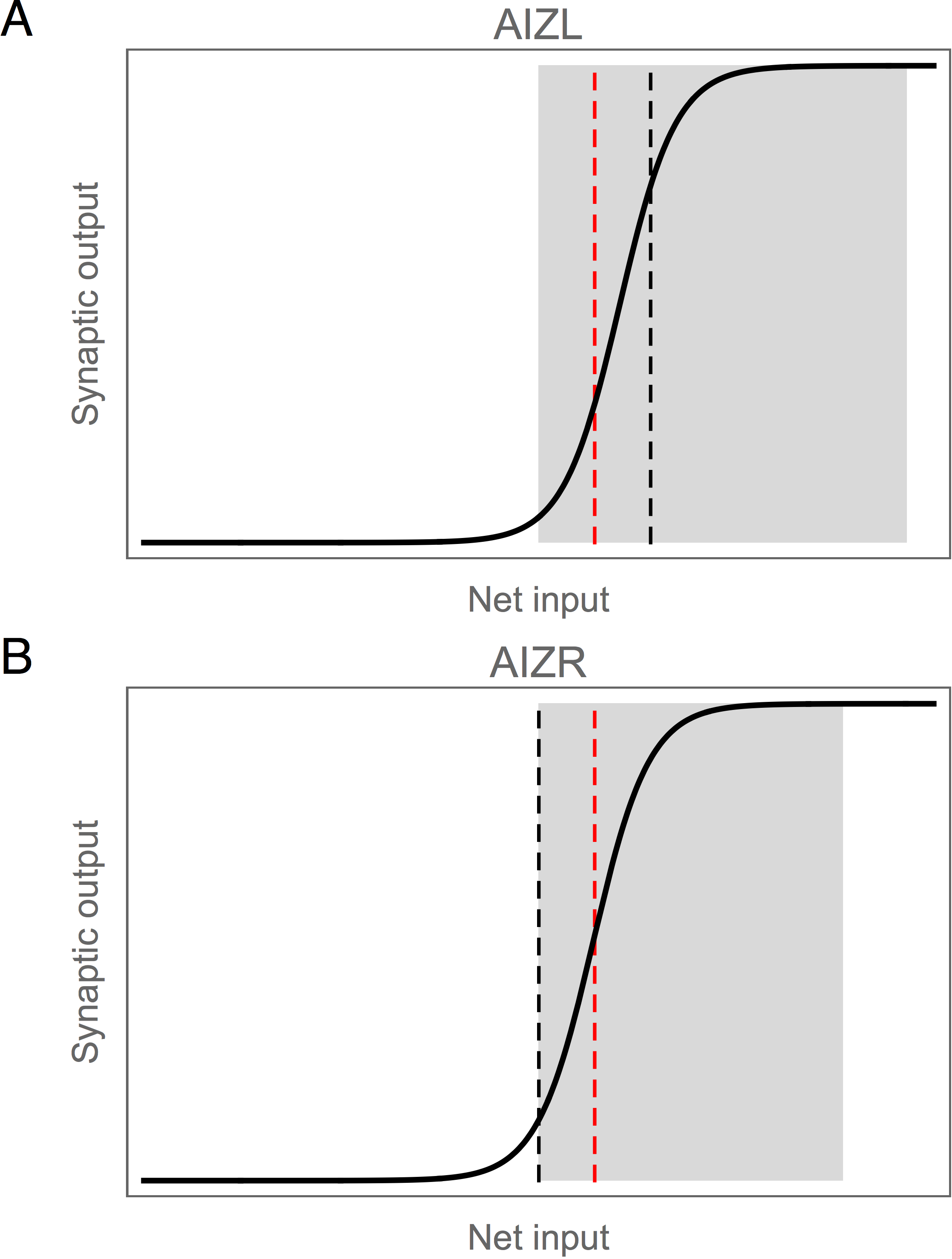

Supplement: S3 Fig — Synaptic transfer functions for the left (A) and right (B) AIZ cells (solid black). Resting potential of the cells before effects from the gap junction shown with dashed black line. Resting potential of the cells after equalization from the gap junction exchange shown with red dashed line. AIZ cells have incoming excitatory chemical synapses from AIY cells, left and right respectively. Therefore, activity in AIYR/AIYL drives the inputs to AIZR/AIZL into the gray region, respectively. The relative strength of the connections are shown by the size of the region. However, because AIYR shows very little activity, changes in membrane potential in AIZR are not due to the chemical synapse; instead they are due to changes in the membrane potential of AIZL through the gap junction. Unlike in AIY, the balance of the resting potential and the sensitive region of the synaptic transfer function in both AIZ cells results in a response to changes in concentration to negative and positive changes in concentration. (TIF) [file pone.0140397.s003.tif]

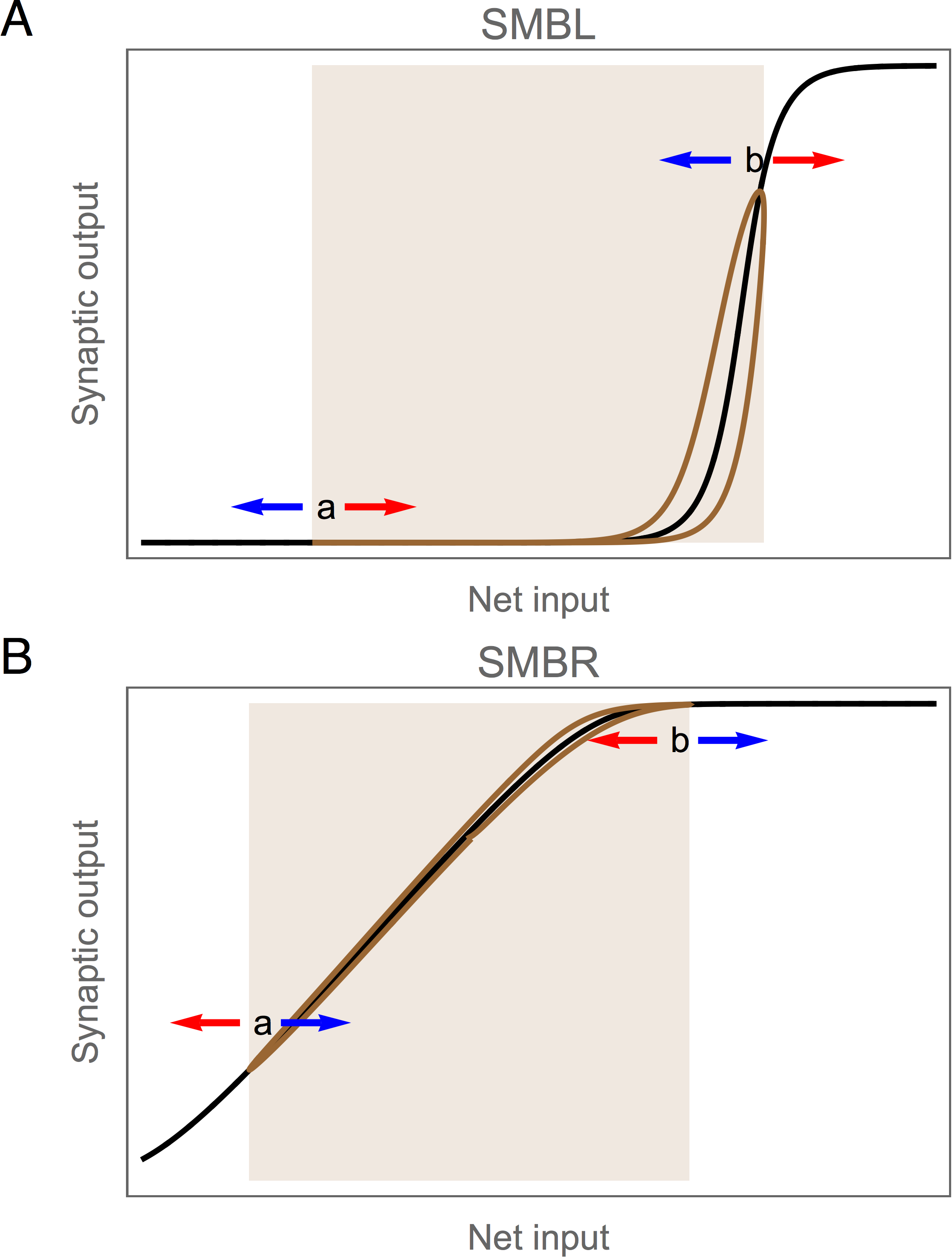

Supplement: S4 Fig — Synaptic transfer functions for the left (A) and right (B) pair of dorsal and ventral motor neurons, SMBL and SMBR, respectively (black trace). Instantaneous synaptic output as a function of net input when the head sweep oscillation is present (brown trace). Shaded areas show the range of oscillation due to the incoming connections from the pattern generator. For each of the SMB pairs, the input to the dorsal and ventral cells moves out of phase over the brown trajectory. As a result, when the dorsal motor neuron is at point a in the curve, the ventral motor neuron is at point b, and vice versa. Red and blue arrows show the effects of negative and positive changes in concentration on the input to the motor neurons, respectively. Due to the shift in the biases for the synaptic transfer functions, a change in concentration sometimes results in a change in the dorsal but not the ventral synaptic output, and viceversa. For the SMBL pair (A), a change in concentration results in a change in the synaptic output of the neuron in b, but not of the neuron in a. For the dorsal/ventral SMBR pair (B), a and b represent the opposite regions: the neuron at a is more sensitive to changes in input than the other neuron at d. To different degrees, the same is the case for other points along the curve. In both pairs of SMB neurons, the result is an antiphase dorsal/ventral gating mechanism. (TIF) [file pone.0140397.s004.tif]
